# SARS-CoV-2-induced cytokine storm drives prolonged testicular injury and functional impairment in mice that are mitigated by dexamethasone

**DOI:** 10.1371/journal.ppat.1012804

**Published:** 2025-01-07

**Authors:** Stefanos Giannakopoulos, Jin Pak, Jackson Bakse, Monika A. Ward, Vivek R. Nerurkar, Michelle D. Tallquist, Saguna Verma

**Affiliations:** 1 Department of Cell and Molecular Biology, John A. Burns School of Medicine, University of Hawaii at Manoa, Honolulu, Hawaii, United States of America; 2 Department of Tropical Medicine, Medical Microbiology and Pharmacology, John A. Burns School of Medicine, University of Hawaii at Manoa, Honolulu, Hawaii, United States of America; 3 Institute for Biogenesis Research, John A Burns School of Medicine, University of Hawaii at Manoa, Honolulu, Hawaii, United States of America; 4 Center for Cardiovascular Research, John A. Burns School of Medicine, University of Hawaii at Manoa, Honolulu, Hawaii, United States of America; University of Virginia, UNITED STATES OF AMERICA

## Abstract

Compromised male reproductive health, including reduced testosterone and sperm count, is one of the long COVID symptoms in individuals recovering from mild-severe disease. COVID-19 patients display testicular injury in the acute stage and altered serum fertility markers in the recovery phase, however, long-term implications on the testis remain unknown. This study characterized the consequences of SARS-CoV-2 on testis function. The K18-hACE2 mice that survived SARS-CoV-2 infection were followed for one month after infection and the testicular injury and function markers were assessed at different stages of infection and recovery. The long-term impact of infection on key testes function-related hormones and male fertility was measured. The efficacy of inflammation-suppressing drug in preventing testicular injury was also evaluated. The morphological defects like sloughing of spermatids into the lumen and increased apoptotic cells sustained for 2–4 weeks after infection and correlated with testicular inflammation and immune cell infiltration. Transcriptomic analysis revealed dysregulation of inflammatory, cell death, and steroidogenic pathways. Furthermore, reduced testosterone levels associated with a transient reduction in sperm count and male fertility. Most testicular impairments resolved within one month of infection. Importantly, dexamethasone treatment attenuated testicular damage, inflammation, and immune infiltration. Our results implicate virus-induced cytokine storm as the major driver of testicular injury and functional impairments, timely prevention of which limits testis damage. These findings serve as a model for evaluating therapeutics in long COVID patients and may guide clinical strategies to improve male reproductive health outcomes post-SARS-CoV-2 infection.

## Author summary

Hypogonadism in males with long COVID is a concern because of the high re-infection rates. Establishing animal models to define testicular injury mechanisms is key to evaluating therapeutic candidates for the prevention of testis function decline. We show that K18-hACE2 mice mimic many features of compromised male reproductive health seen in long COVID patients including a decrease in testosterone levels and male fertility. The findings reveal SARS-CoV-2-induced systemic inflammation as the underlying cause of prolonged testicular damage rather than direct virus replication that can be mitigated by dexamethasone treatment. Data provides evidence that fertility impairments are transient, supports timely treatment with inflammation-suppressing drugs to prevent testis injury, and will guide clinicians in monitoring male reproductive health and treatment decisions.

## Introduction

The coronavirus disease 2019 (COVID-19) pandemic caused by the severe acute respiratory syndrome coronavirus 2 (SARS-CoV-2) remains a major health concern even with new therapies and vaccinations that have diminished acute fatality rates. A wide range of multi-organ chronic symptoms in recovering patients, also called post-acute sequelae of infection (PASC) or long COVID, contribute to debilitating conditions that affect millions worldwide [1–3]. The incidence of long COVID is estimated at 10–30% of non-hospitalized cases, including asymptomatic infections, 50–70% of hospitalized cases, and 10–12% of vaccinated cases, with the highest percentage of diagnoses between the ages of 36 and 50 years [4,5]. A compromised male reproductive health is now included as one of the symptoms of long COVID [4]. Strong clinical data report orchitis as one of the common symptoms during the symptomatic stage of COVID-19 [6–8]. The postmortem analyses of testes from COVID-19 patients revealed signs of testicular pathology, including tubular injury, germ cell and Leydig cell (LC) depletion, and leukocyte infiltration [7,9–11]. The presence of SARS-CoV-2 RNA in the testis and semen is a rare event [12,13]. However, alterations in male fertility parameters like decreased testosterone levels and impairment of sperm parameters, including reduced concentration and motility, are reported for months after recovery in all aged COVID-19 patients, including reproductive-aged (20–49 yrs) men [14–16]. Notably, patients recovering from both severe and moderate/mild COVID-19 symptoms display statistically significant impairment of sperm parameters for months after recovery [16–20]. A recent study highlighted that 55% (n = 66) of COVID-19 patients exhibited hypogonadism during their 7-month and 12-month follow-up [17,18]. However, our understanding of the kinetics and mechanisms underlying testicular injury and impaired function beyond the analysis of semen parameters is limited.

The testicular immune environment is tightly governed by an elaborate communication network between different resident cells, including Sertoli cells (SC), that nurse undifferentiated spermatogonial stem cells (SSC) and form the blood-testis barrier (BTB), and LC required for maintaining spermatogenesis and local immune homeostasis [21]. Since ACE2 is highly expressed in human testicular cells, it was speculated that SARS-CoV-2 can establish productive infection in the testicular cells. However, recent studies demonstrated that SARS-CoV-2 replication was either limited in human testis explants [22] or did not lead to productive infection in organoids [23] suggesting that the testicular injury is more likely a bystander effect of systemic inflammation. While testis injury has not been characterized in the mouse-adapted SARS-CoV-2-infected mice [24], a recent study detected low levels of reporter SARS-CoV-2 nanoluciferase virus by bioluminescence imaging in the testis of K18-hACE2 mice [25]. We recently showed that K18-hACE2 mice, where the hACE2 receptor is predominantly expressed in lung epithelial cells, displayed testicular injury during the acute stage of the infection in the absence of active virus replication that mimics injury features observed in humans [22].

Although clinical studies provide strong evidence of impaired fertility markers for months in recovering patients [16–19,26–28], many critical questions regarding mechanisms underlying testicular pathogenesis and the impact on testis function and male fertility remain unclear. These data can be only generated using an animal model. The spermatogenesis cycle of the development of male germ cells in the seminiferous tubules takes ~9 days to complete in mice. Therefore, we here followed the survivor K18-hACE2 mice for up to one month after infection, which is the approximate time required for three spermatogenesis cycles, to characterize the long-term effects of systemic inflammation on testicular injury markers and functional impairments. Survivor mice displayed sustained testicular injury, inflammation, and infiltration of immune cells post-acute infection after the virus was cleared from the lungs. Transcriptomics analysis indicated sustained dysregulation of key pathways associated with testis immune homeostasis, LC function, and cell death. Further, fertility marker assays indicated a sustained decrease in testosterone levels, which correlated with a short-term reduction in sperm counts and fertility rates in recovering males. However, most injury markers and defects, except lower testosterone levels, resolved within 4 weeks of infection. Notably, corticosteroid treatment limited the severity of the testicular injury suggesting that systemic cytokine storm is central to the testicular injury, suppression of which might prevent long-term functional impairments in the long COVID patients.

## Results

### Morphological alterations in the testis of SARS-CoV-2-infected K18-hACE2 mice

**Fig 1:**
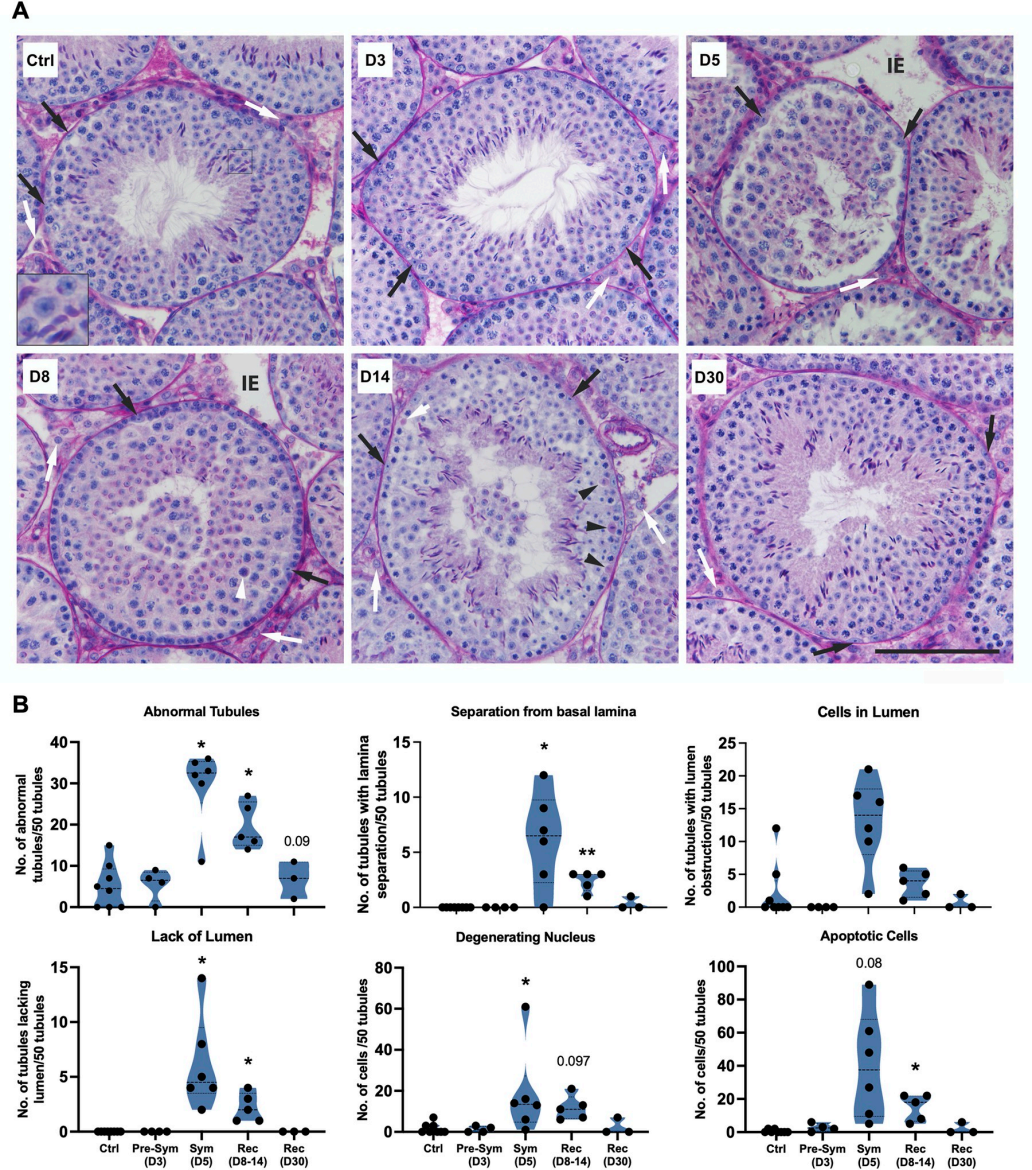
Testis morphological defects in SARS-CoV-2-infected K18-hACE2 mice. The days post-infection (D) are represented as pre-symptomatic (D3), symptomatic (D5), short-term recovery (D8-14), and long-term recovery stages (D30). **(A)** Representative images of PAS-H-stained testis sections from mock (Ctrl) and infected mice. Leydig cells, white arrows; Sertoli cells, black arrows. Inset shows normal round and elongating spermatids. Various testicular abnormalities were observed, including the occurrence of apoptotic germ cells (white arrowheads in D8), apoptotic meiotic cells (black arrowheads in D8), cells with degenerating nuclei (short white arrow in D8), tubules lacking clear lumen and with a separation of germ cell layer from the basal membrane (D5), and with the sloughing of healthy spermatids and spermatocytes into the lumen (D8-14). The stages of seminiferous epithelium are: II-IV (D30), V-VI (Ctrl & D3) VII-VIII (D5 & D8), X-XII (D14). Inset, 3x magnification. Scale, 100 μm. **(B)** Quantification of seminiferous tubule organizational and germ cell defects. Each data point corresponds to a different mouse. For each male, 50 tubules were examined. Statistical significance (t-test, Ctrl vs. Infected). *p<0.05 and **p<0.01. The differences approaching significance (p = 0.05–0.1) are shown directly in the graph.

To assess the kinetics of testicular injury in the post-acute stage of SARS-CoV-2 infection, we examined survivor K18-hACE2 mice (20% survival rate, S1A Fig) for up to 30 days post-infection (D30). The mice lost body weight drastically during the acute stage of the disease, and their recovered body weight in the survivors remained lower than the controls (S1B Fig). The virus was cleared from the lungs by D8, and as expected, no infectious virus was detected in the testis at any time point (S1C–S1E Fig). The testicular histology was evaluated when viral replication can be detected in the lung (D3), at the peak symptomatic stage (D5), short-term recovery stage (D8, D14), and long-term recovery stage (D30, approximate days for spermatogonia to differentiate into spermatozoa). The seminiferous tubules were analyzed for specific defects as described by us before [29]. Testes from control mice showed normal spermatogenesis, with the germ cell types and tubular organization as expected. At D3, the testes resembled those of control mice (Fig 1A). The support cells in the interstitial space (LC) and seminiferous epithelium (SC) looked normal in controls and D3. During the peak symptomatic stage and short-term recovery stage, interstitial edema and various tubular defects including separation of germ cells from the basal lamina and premature germ cell sloughing to the lumen were observed (Fig 1A). In addition to the assessment of tubule organization, individual germ cells were scored for their normalcy (Figs 1 and S2A). Germ cell abnormalities were observed in symptomatic and short-term recovery stages, including cells with degenerating nuclei (Fig 1A, D5) or undergoing apoptosis (Fig 1A, D8-14), and other cellular defects (S2 Fig). Germ cell and seminiferous epithelium morphology returned to normal by D30 (Fig 1A). Quantification of testicular defects shown in Figs 1B and S2 support that testicular pathological alterations persist for at least 2 weeks post-SARS-CoV-2 infection.

### Gene expression analysis of testis post-SARS-CoV-2 infection

**Fig 2:**
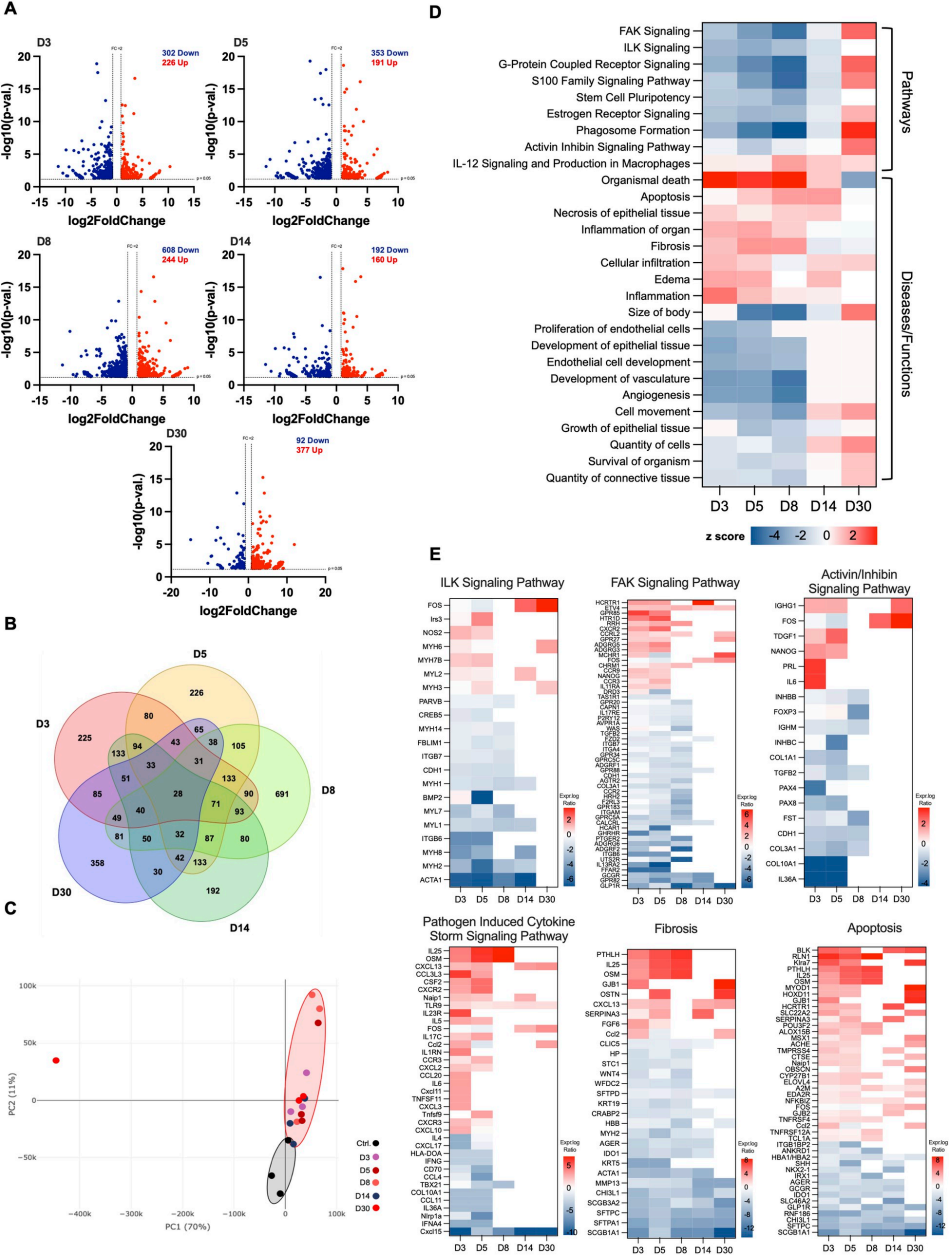
RNA-seq analysis of the testis identifies dysregulation of distinct transcriptional pathways at different stages of SARS-CoV-2 infection. **(A)** Volcano plots of differentially expressed genes (DEGs) (n, 3 mice per time point) at indicated time points, dotted lines represent cutoff: p< 0.05 and log2fc > |1|, genes increased after infection, red; and genes decreased after infection, blue. **(B)** Venn diagram of total DEGs at D3 (red), D5 (yellow), D8 (light green), D14 (dark green), and D30 (blue). **(C)** Principal component analysis (PCA) performed using DEGs (p <0.05 and log2fc > |1|) and normalized transcripts per million (controls, black circle; infected, red circle) **(D)** Heatmap of top pathways and select diseases and functions revealed by Ingenuity Pathway Analysis (IPA), filtered to remove erroneous results (upregulated, red; downregulated, blue). **(E)** The expression log2 ratio of upregulated (red) and downregulated (blue) genes associated with ILK, FAK, and activin/inhibin signaling pathways and pathogen-induced cytokine storm, fibrosis, and apoptosis signaling pathways as determined by IPA in each dataset (p< 0.05; log2fc > |1|) at indicated time points.

To gain insights into the impact of SARS-CoV-2 at the molecular level, we examined the transcriptomic signatures in the testes by RNA-seq analysis at D3, D5, D8, D14, and D30. A total of 1,865 non-redundant differentially expressed genes (DEGs) were identified across all time points (p<0.05). As shown in the Volcano plots and the Venn diagram (Fig 2A and 2B), the highest number of DEGs were observed at D8 (691) while most upregulated genes were observed at D30 (377). Principal component analysis separated the control testis and the infected samples (Fig 2C). Gene ontology (GO) overrepresentation analysis (ORA) of DEGs at earlier time points revealed the downregulation of genes associated with cell adhesion and extracellular structure reorganization while genes associated with cytokine and chemokine secretion and lymphocyte differentiation pathways were mostly upregulated in short-term recovery stage (S3A and S3B Fig). The most prominently upregulated pathway at D30 was the response to wounding and wound healing (S3C Fig).

IPA analysis at early time points revealed that focal adhesion kinase (FAK) and integrin-linked kinase (ILK) signaling pathways, which play an important role in the formation of tight junctions of the blood-testis barrier (BTB) and cell-cell adhesion [30,31] were decreased (Fig 2D). Genes in signaling pathways associated with stem cell pluripotency, estrogen receptor, and phagosome formation were decreased at all time points except D30, while the activin-inhibin pathway was transiently downregulated only at D5. The IPA analysis of select diseases and functions revealed significant upregulation of organismal death, cellular infiltration, apoptosis, necrosis, inflammation, IL-12 signaling, and fibrosis at most of the time points. Individual DEGs of select pathways/functions are shown in Fig 2E. The ILK signaling pathway includes the downregulation of ACTA1, a skeletal muscle α-actin gene that plays a role in muscle contraction and cytoskeleton organization [32]. INHBB and TGFB2, are both involved in activin/inhibin signaling pathways and these genes were significantly downregulated only at early time points. Transcript of Oncostatin-M (OSM), which is a multi-functional cytokine that promotes inflammation [33] and negatively regulates LC differentiation [34] was significantly upregulated and was part of pathogen-induced cytokine storm, fibrosis, and apoptosis pathways. Several cytokines and chemokines including IL25, CXCL13, CSF2, CXCL2, CCL2, Naip1, and TNF family members were identified to be upregulated, however, CXCL15, shown to suppress proliferation and expansion of macrophage progenitors, was significantly downregulated at all time points. These data indicate that SARS-CoV-2 infection can impact multiple pathways associated with testicular function and immune homeostasis in the acute and short-term recovery stages. However, genes associated with the apoptotic pathway remain impaired until D30.

Next, upstream regulator analysis revealed that the androgen receptor (AR) and angiotensinogen (AGT) which play an important role in testicular growth/differentiation [35], were downregulated in the early time points, suggesting LC dysfunction during the acute and short-term recovery stage of the disease (S4A Fig). Further, important transcription factors involved in steroidogenesis, GATA1, GATA2, and GATA6 [36–38] were downregulated with GATA6 downregulated even at D30 (S4A Fig). Interestingly, genes SFTPC, SFTPA1, and SCGB1A1 regulated by GATA6 were also decreased at all the time points (S4B Fig). Similarly, CYP2J13, TNNT3, and PVALB genes targeted by the androgen receptor (AR) were also downregulated at most of the time points (S4C Fig). In contrast, mediators of apoptosis THZ1, a CDK7 inhibitor, fibrosis (SIX1), and inflammation (TNFSF12) were upregulated in the early time points.

### Testicular inflammatory cytokine levels correlate with systemic inflammation during acute and post-acute stages of infection

**Fig 3:**
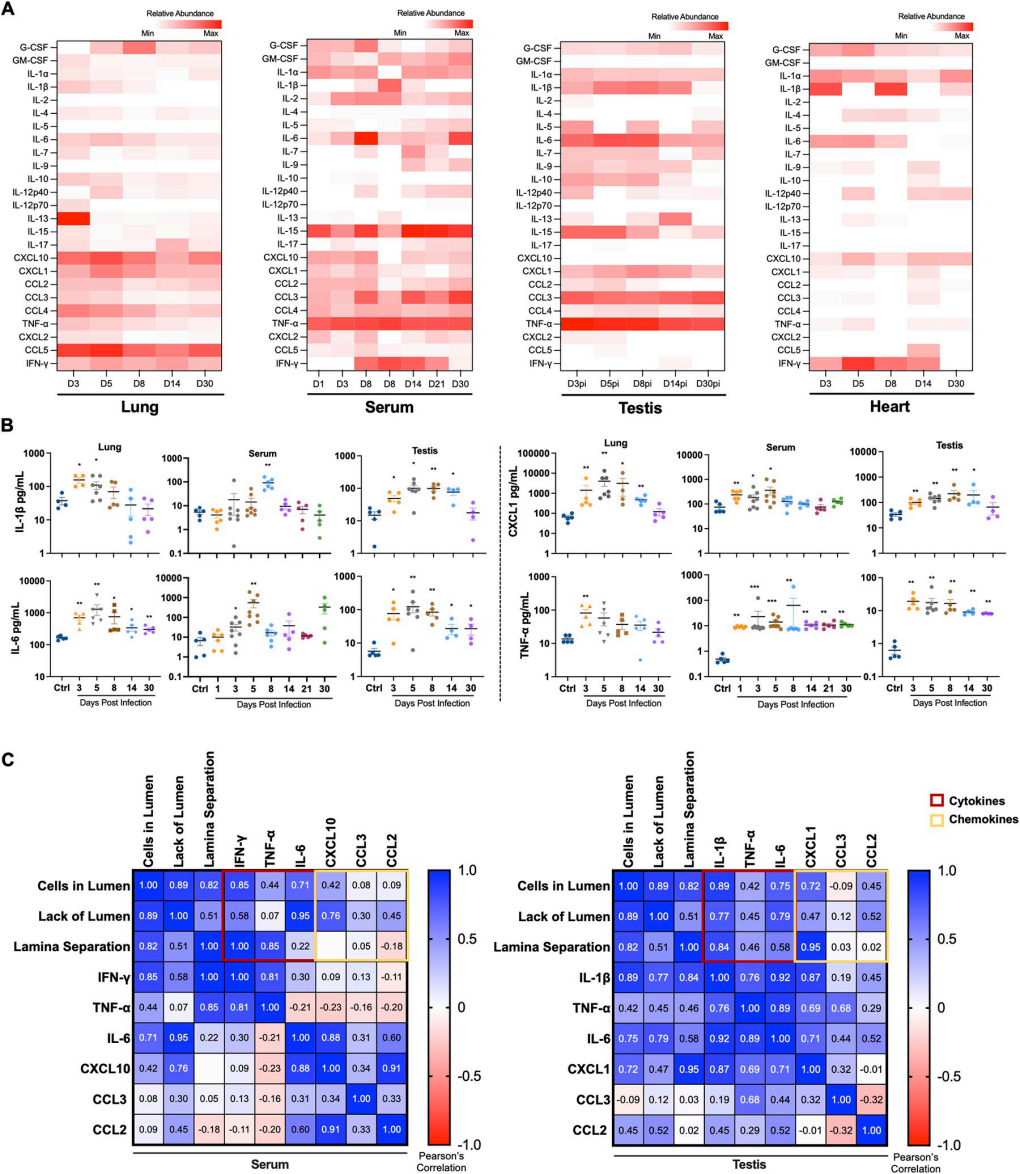
Sustained increase in the levels of pro-inflammatory cytokine and chemokine in the testis correlate with systemic inflammation. **(A)** Heatmap of a panel of COVID-19 specific cytokine and chemokines measured using LUMINEX assay in the tissue homogenates (lung, testis, and heart) and serum of K18-hACE2 mice at indicated time points post-infection (n, *≥*4 mice per group). Data were normalized to the control, log10 transformed, and expressed as relative abundance (red, increased; blue, decreased; and white represents unchanged compared to uninfected controls). **(B)** Levels of IL-1β, IL-6, CXCL1, and TNF-α expressed as pg/mL. Individual data points represent different mice. The statistical significance (*p<0.05, **p<0.01, ***< p<0.001) was determined using Mann-Whitney test for all analyses **(C)** Pearson’s correlation coefficient analysis between testicular defects (cells in lumen, lack of lumen, and lamina separation) and key cytokines (red box) and chemokines (yellow box) in the serum (left) and testis (right) at D3, D5, and D8. Pearson coefficient >0.6 was considered a significant correlation.

To validate the RNA-Seq data and to understand the correlation between systemic and testicular inflammation with testicular injury, we measured a panel of cytokines and chemokines in the serum and lysates from the lung, testis, and heart. As expected, levels of G-CSF, IL-1β, IL-6, CXCL1, CCL3, and TNF-α increased significantly in lungs, and serum at D3-D14. However, these cytokines and chemokines were also significantly elevated in the testis, and IL-6 and TNF-α levels remained significantly higher even at D30 suggesting that testicular cytokines may take longer to completely resolve (Fig 3A and 3B). To examine the correlation between the cytokines in the serum and testis with gross morphological alterations observed in Fig 1B, we performed Pearson correlation analysis. The correlation between different testicular abnormalities (basal lamina separation, lack of lumen, and cells in lumen) with testicular cytokines IL-1β, IL-6, and TNF-α (Fig 3C right panel) was much stronger than with serum cytokines. These data highlight that the testicular immunosuppressive environment is compromised for weeks after infection and shifts towards a robust proinflammatory response during the acute and short-term recovery stage of the disease.

### Sustained apoptotic cell death in the testis is accompanied by a transient increase in infiltration of immune cells and loss of BTB proteins

**Fig 4:**
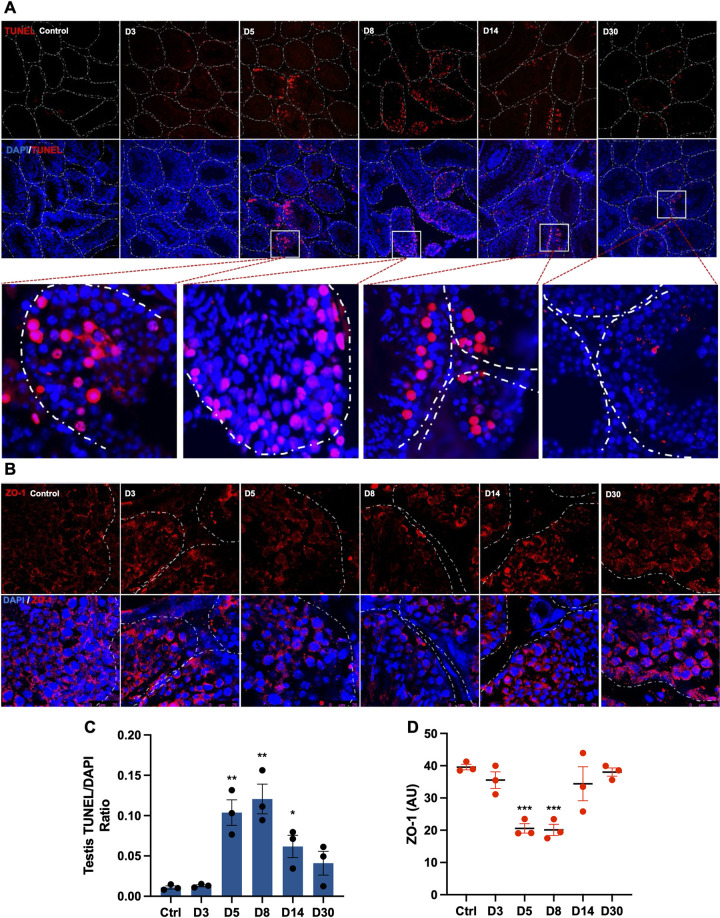
SARS-CoV-2 infection results in sustained apoptotic cell death in the testis. Representative images of **(A)** TUNEL staining and **(B)** ZO-1 staining in testis from the indicated time point. Nuclei were visualized using DAPI stain (blue), and dotted lines represent the shape of individual seminiferous tubules. ImageJ analysis of **(C)** TUNEL staining normalized to respective DAPI positive cells and **(D)** ZO-1staining intensity. Each data point is the average of counts from 3 different fields from each testis section (n, 3 mice/time point). Significance (*p<0.05, **p<0.01, ***p<0.001) was determined using a student’s t-test.

To address the consequence of the pro-inflammatory response in the testis, we assessed cell death at different time points (Fig 4A). TUNEL staining of testis from control mice was comparable to that of D3, however at D5, a significant increase in TUNEL-positive cells was observed, primarily in the cells within the seminiferous tubules. Apoptotic cells in both seminiferous epithelium and in the lumen peaked at D8. While there was a marked reduction in the TUNEL-positive cells at D14 and D30, they remained higher than the control testis, indicating that the seminiferous tubule injury had not resolved completely by D30 (Fig 4C). We next used the relative expression of testicular cell markers in our RNA seq data as a proxy to determine which cell type is predominantly affected during SARS-CoV-2 infection. The cell type proportions in the testis estimated at each time point using CIBERSORTx deconvolution analysis showed a marked decrease in the proportion of the SSCs and SCs following infection compared to uninfected controls. While the SC proportion recovered by D8, the SSC remained low even at the late recovery stages (D14 and D30, S5A Fig). Validation of results from CIBERSORTx analysis using qRT-PCR analysis of cell type-specific marker genes of Leydig cells (STAR, CYP11A1), SC (GATA4, SOX9), and spermatogonia (ZBTB16, SOX2) also indicated a significant reduction in SSC markers till D14 (S5B–S5D Fig) thus correlating with the presence of apoptotic cells inside the seminiferous tubules. Further, the expression of BTB marker ZO-1 was found to be markedly reduced at D5 and D8 (Fig 4B and 4D) suggesting that testicular inflammation may transiently affect the BTB integrity. The interrogation of other genes of gap junction and tight junction families identified the downregulation of multiple genes in the claudin family at both D5 and D8 (S5E Fig). While our testis histology and RNA-seq data indicated that injury markers and dysregulation of most pathological pathways resolved by D30, we also applied a second-degree polynomial regression model to extrapolate the gene expression at D40, D60, and D90 (p<0.05, log2fc |1|, S6 Fig). We observed a complete reversal in the canonical pathways, including ILK and FAK signaling (S6A–S6C Fig) as well as upstream regulators (S6D Fig), further corroborating that testicular injury-related transcriptomic alterations resolve within 4 weeks of infection.

**Fig 5:**
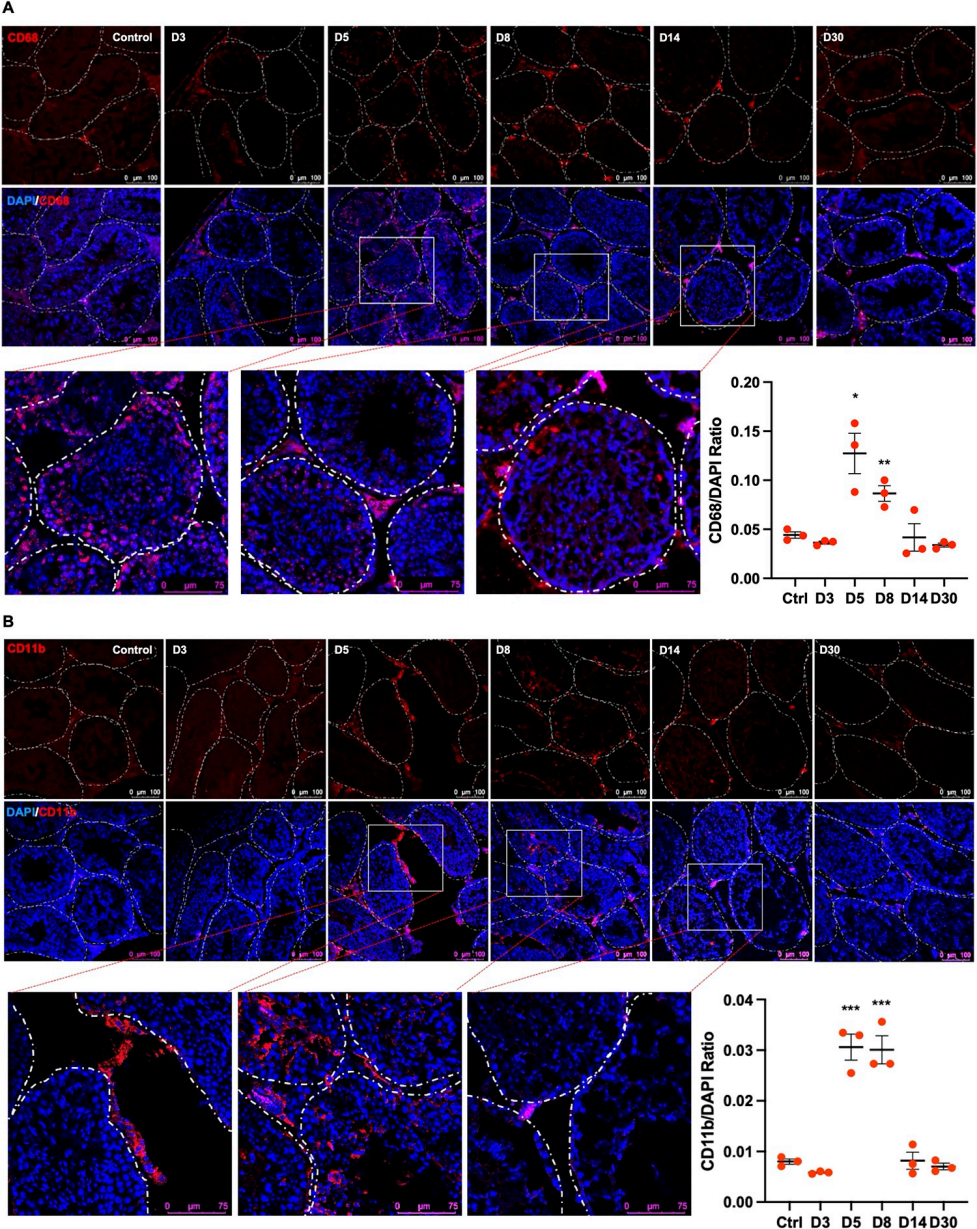
SARS-CoV-2 infection leads to a transient increase in the infiltration of CD68+ and CD11b+ cells in the testis. Representative images of testis sections at indicated days after infection (D). The DAPI (blue) and infiltrating leukocytes were visualized using **(A)** CD68, and **(B)** CD11b markers. Dotted lines outline the shape of individual seminiferous tubules and ImageJ analysis of CD68 and CD11b staining normalized to DAPI-positive cells was done using particle analyzer tool. Each data point is the average of counts from 3 different fields from each testis section (n, 3 mice/time point). Images were taken using Leica SP3, DMI8 confocal microscope. Significance (*p<0.05, **p<0.01, ***p<0.001) was determined using the student’s t-test.

Pro-inflammatory chemotactic factors such as CXCL1 and CCL3 are directly associated with increased tissue immune cell infiltration, therefore, we next examined lymphocyte infiltration in the testis. The staining for the CD68 (Fig 5A) was comparable between controls and D3 testis, however, a significant increase of CD68-positive cells both in the interstitial space and inside the seminiferous tubules was observed at acute and short-term recovery stages. Similarly, staining of CD11b revealed a high number of positive cells at D5 and D8 (Fig 5B). The incidence of both CD68+ and CD11b+ cells was comparable to the control group at D14 and D30. Collectively these data suggest that SARS-CoV-2 infection leads to sustained cell death in the testis while infiltration of immune cells is one of the short-term effects that correlates with loss of BTB proteins.

### Testicular function is compromised in SARS-CoV-2-infected K18-hACE2 mice

**Fig 6:**
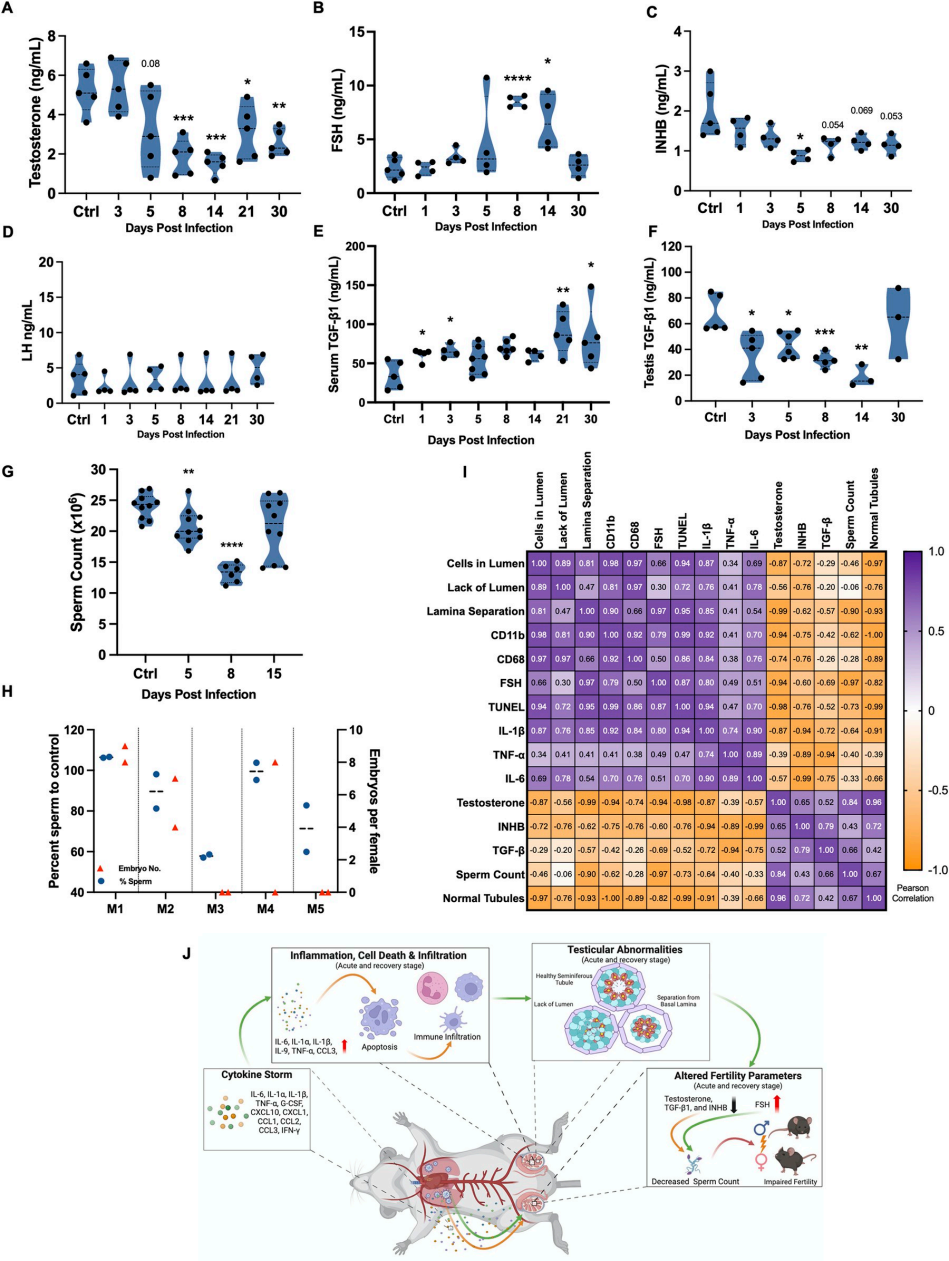
Consequences of SARS-CoV-2 infection on testis function. Serum levels of **(A)** testosterone, **(B)** follicle-stimulating hormone (FSH), **(C)** inhibin B (INHB), and **(D)** luteinizing hormone (LH) were measured using ELISA at indicated time points and expressed as ng/mL serum (n, at least 4 mice per group). TGF-β levels in **(E)** serum and **(F)** testis homogenates were measured using ELISA and expressed as ng/mL at indicated time points post-infection. **(G)** Sperm count per epididymis was measured at indicated time points (n, 3–5 mice per group). Each data point represents data from one epididymis. **(H)** Age-matched male survivors were mated with females (2 females per male) for 6 days and then separated. The males were sacrificed after 6 days to measure the sperm count while pregnancy was evaluated after 10 days by counting the embryos. The percent change in the sperm count/epididymis (left y-axis, circle, blue) in each male as compared to un-infected controls and the number of fetuses (right y-axis, triangle, red) in each female (n, 2 females for each male). **(I)** Pearson’s correlation coefficient analysis of testicular defects, pro-inflammatory and cell death markers, and fertility parameters during the acute and short-term recovery stages (D3, D5, and D8). Significance (*p<0.05, **p<0.01, ***p<0.001, ****p<0.0001) was determined using student’s t-test. **(J)** Schematic illustration of the proposed events leading to testicular injury generated using BioRender.com. SARS-CoV-2 infection increases serum levels of pro-inflammatory cytokines/chemokines that trigger testicular inflammation leading to downstream effects including loss of BTB integrity, infiltration of immune cells, and apoptosis. These events manifest in the disruption of the morphology of the seminiferous tubules and impaired testicular function marked by hormone dysregulation and lower sperm count. Most of these impairments resolve within two spermatogenesis cycles, however, depending on the severity of these impairments, infection may lead to a short-term decrease in fertility in mice recovering from acute infection.

The observation of gross testicular abnormalities and inflammation led us to another important question—what is the effect of these impairments on testicular function? To address this, we assessed the serum levels of testosterone, follicular stimulating hormone (FSH), inhibin B (INHB), and luteinizing hormone (LH). Serum testosterone levels declined significantly during the short-term recovery stage. Even though they increased slightly at D21 and D30, they remained significantly lower than that of the control (Fig 6A). The serum FSH levels were significantly elevated compared to controls at D8 and D14 (Fig 6B) while LH and INHB levels either remained unaffected or declined transiently (Fig 6C and 6D). Considering the important role TGF-β plays in testis function including steroidogenesis and SC tight junction dynamics, we next measured serum and testicular levels of TGF-β1 (Fig 6E and 6F). While serum TGF-β1 levels increased after infection, resembling what has been shown in human COVID-19 patients [39], we observed a gradual decline in testicular TGF-β1 levels with the lowest levels seen at D14, following which they recovered by D30.

Finally, the analysis of epidydimal sperm count and fertility was performed to test the downstream effect of these alterations. Interestingly, a decline in total numbers of epidydimal sperm count was observed not only at the acute stage but also during the short-term recovery phase (~50% reduction in the sperm count at D8, Fig 6G). To evaluate if decreased sperm count affected the ability to reproduce, the fertility was tested for a subset of the recovered mice first at D9 when the mice completely recovered from symptoms and displayed significant improvement in body weight (S1B Fig). Five survivor males, who were proven fertile before the infection, were paired with 2 females each at D9 for 6 days followed which they were sacrificed for epididymal sperm analysis to correlate with pregnancy outcome. Mating was confirmed in all females by observing a vaginal plug within 2–5 days. A week after separation from the males, the pregnancy was scored by counting the number of embryo implantation sites and was observed in females paired with only 3 out of 5 males. Females paired with the males with a reduction in the sperm count by more than 30% did not get pregnant (Fig 6H). A similar fertility assay was also performed on three survivor mice at D14 when a trend in the recovery of sperm count was seen (Fig 6G). All three males were found to be fertile, as evidenced by at least one female paired with each mouse becoming pregnant (S7 Fig), suggesting that the impact on fertility of recovering males is only in the early phase of recovery when the sperm count is significantly low. Pearson’s correlation matrix analysis of different injury and function markers revealed that the testicular defects, immune cell infiltrates, cell death, and testicular cytokines have a strong positive correlation with each other (Fig 6I). In contrast, a strong inverse relationship was observed between the testis injury and function markers including serum testosterone and inhibin B levels, sperm count, and testicular TGF-β levels (Fig 6I).

### Corticosteroids mitigate testicular damage and reduce immune infiltration

**Fig 7:**
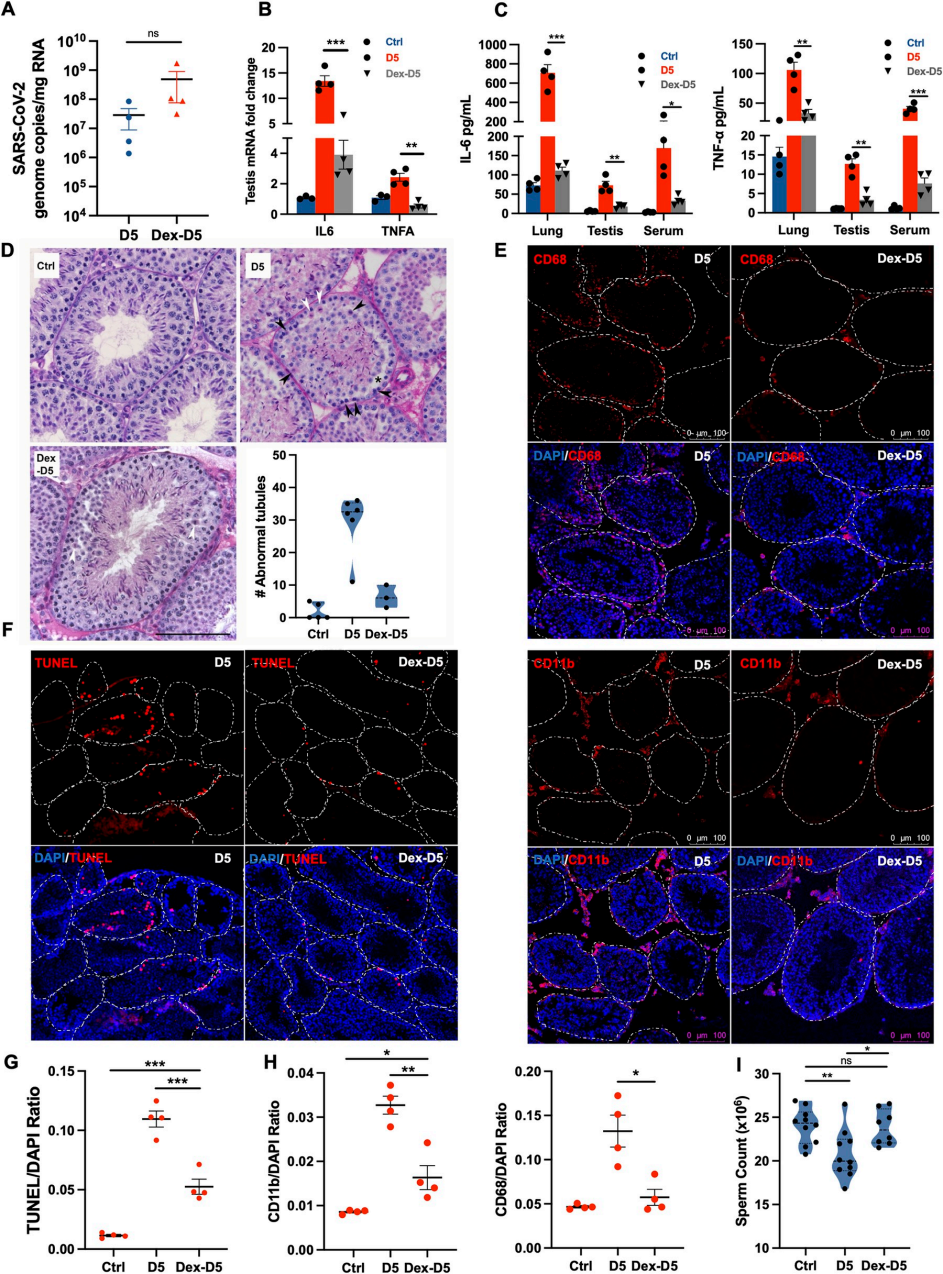
Effect of dexamethasone treatment on the testicular injury in SARS-CoV-2 infected mice. Infected mice were treated with 10mg/kg dexamethasone starting D2 for 3 days and at D5 **(A)** SARS-CoV-2 genome copies in the lung measured using qRT-PCR and expressed as copies/mg RNA. The mRNA fold change of **(B)** IL6 and TNFA gene expression in the testis from infected mice with and without dexamethasone treatment using qRT-PCR at D5 **(C)** IL-6 and TNF-α levels in the lungs, serum, and testis measured using ELISA at D5 **(D)** Representative images of PAS-H-stained testis sections from mock (Ctrl), infected (D5), and dexamethasone-treated (Dex-D5) mice. Various testicular abnormalities were observed including the occurrence of apoptotic germ cells (black arrowheads) and apoptotic meiotic cells (white arrowheads), lack of lumen, separation from the basal membrane (*), and interstitial edema (IE). In Dex-D5 testes few minor defects were observed, exemplified here as 2 apoptotic meiotic cells (white arrowheads). Scale, 100 μm. For quantification of seminiferous tubule organizational and germ cell defects, 50 tubules were examined in each male. Each data point corresponds to a different mouse. **(E)** Representative images of the testis from SARS-CoV-2-infected mice at D5 with or without dexamethasone treatment with nuclei stained using DAPI (blue) and infiltrating leukocytes stained using antibodies against CD68 (top panel; red), and anti-CD11b (bottom panel; red). Dotted lines outline the shape of individual seminiferous tubules. **(F)** Representative images of the testis from mice with or without dexamethasone treatment with nuclei stained using DAPI (blue) and apoptotic cell visualized using TUNEL (red). ImageJ analysis of **(G)** TUNEL and **(H)** CD68 and CD11b staining normalized to DAPI staining measured using particle analyzer tool. Each data point represents the mean of three fields from different testis sections (n, 4 mice per group). Images were taken using Leica SP3, DMI8 confocal microscope. **(I)** Sperm count per epididymis was measured at D5 in the presence or absence of dexamethasone treatment and PBS control (n, 4–5 mice per group). Each data point represents data from one epididymis. Significance (*p<0.05, **p<0.01, ***p<0.001, ****p<0.0001) was determined using student’s t-test.

Clinical use of dexamethasone has been shown to reduce lung pathology and systemic inflammation in both humans and mice [40,41]. Therefore, we used dexamethasone treatment to test if suppression of inflammation during the peak virus replication stage (D2-D5) would prevent testicular pathology in infected mice. SARS-CoV-2 titers in the dexamethasone-treated mice trended higher in the lungs at D5 (Fig 7A) and in agreement with other similar studies [41,42]. As expected, dexamethasone treatment attenuated the levels of IL-6 and TNF-α in the lungs and serum. Interestingly, a significant decline of IL-6 and TNF-α was also observed in the testis at D5 (Fig 7B and 7C). When the testicular pathology was compared in both groups, we observed a marked improvement in the overall testicular morphology. A reduction in the number of abnormal tubules in the drug-treated testis was noted when compared to untreated mice testis (Fig 7D). Other injury markers including tubules with lumen obstruction and sloughing of cells were also decreased in the dexamethasone-treated group when compared to D5 (S5 Fig). Further, infiltrating CD11b+ and CD68+ immune cells and TUNEL-positive cells were also much lower in the dexamethasone-treated group compared to untreated males (Fig 7E and 7F), however, they remained higher than controls (Fig 7G and 7H). Additionally, dexamethasone treatment also prevented the decline in sperm counts that was comparable to the control group at D5 (Fig 7I) These data support the involvement of SARS-CoV-2-induced inflammatory mediators in initiating testicular injury and suggest that timely treatment of anti-inflammatory drugs can prevent testicular injury.

## Discussion

Using survivor K18-hACE2 transgenic mice as a model to study long COVID, we characterized the pathophysiology of testicular injury during the acute and post-acute stages of SARS-CoV-2 infection. Testis from infected mice presented defects, including increased apoptotic germ cells and germ cells with degenerating nuclei and various tubule organization defects, as well as dysregulation of key steroidogenic and immune homeostasis pathways during acute and post-acute stages of infection. These defects are associated with testicular inflammation and infiltration of immune cells into the lumen and resolve mostly within two spermatogenesis cycles. The impairment in the testicular hormones, specifically reduced testosterone levels, persisted for up to four weeks after infection and correlated with a short-term reduction in sperm count and male fertility. The testicular injury in these mice is sensitive to corticosteroid treatment that alleviates major hallmarks of pathology. Collectively, these findings help us decipher the underlying pathophysiology of male reproductive health in patients with long COVID.

Relevant long COVID mouse models that reflect testicular impairments seen in humans are critical for elucidating underlying mechanisms and testing effective preventive therapeutic strategies. Since SARS-CoV-2 infection leads to severe disease in K18-hACE2 mice, they more accurately mimic male health complications in patients mainly with moderate to severe disease observed in humans [43]. Other mouse models developed to study SARS-CoV-2 pathogenesis include mouse-adapted SARS-CoV-2 strain [24,44] and the humanized mouse model, MISTRG6 mice [41], but testicular injury has not been reported in these mice so far. However, our data aligns with some of the findings reported in the golden Syrian hamster model where SARS-CoV-2 infection led to testicular damage and decreased sperm count [45]. However, the limitation of our study is that our data does not accurately recapitulate the impact on the testis function seen in mild or asymptomatic cases who are also at risk of developing long COVID though the risk is slightly lower compared to patients with severe disease [4,5]

Our study extends the histopathological changes observed in post-mortem testicular tissue from COVID-19 patients [7,10] and describes the specific type of tubular defects observed during different stages of infection and recovery. So far, the long-term impact of infection on male reproductive health has been assessed using only serum and/or semen. Our data provides strong evidence for the persistence of specific testicular defects like sloughing of immature germ cells into the lumen during the early recovery stages. These defects are also characteristic hallmarks of testicular injury also seen in autoimmune orchitis and testicular cancer [46,47]. Persistent inflammation is a hallmark of long COVID patients [48,49]. While many inflammatory cytokines like IL-1β, IL-6, and TNF-α were elevated in the testis and serum, interestingly, cytokines that modulate the testicular macrophage phenotype like IL-13 and IL-10 were only induced in the testis. IL-6 and TNF-α alone have been shown to induce testicular germ cell death [50] and affect the integrity of the BTB [51,52]. Our data suggests that an increase in the testicular cytokines and chemoattractant CXCL1 may directly impact infiltration of macrophages and BTB integrity as also evidenced by the dysregulation of pathways involved in tissue repair like FAK and ILK signaling.

The presence of cell death within the seminiferous tubules for up to 4 weeks after infection is an important finding and suggests that complete recovery may take longer than one spermatogenesis cycle, which is 9 days in mice compared to 72 days in humans [53]. One reason for the delayed clearance of TUNEL-positive cells can be the timely restoration of BTB integrity that may prevent access of macrophages in the lumen as seen in our data. Further, sustained decrease in testosterone levels along with the downregulated steroidogenesis genes (GATA6 and GATA1) indicate a long-term impact on LC function and aligns well with clinical data showing decreased testosterone levels in COVID-19 patients for up to 7 months after recovery [18,54,55]. Increased FSH levels are also indicative of abnormal spermatogenesis and may suggest primary testicular failure due to its role in the feedback mechanism via the HPG axis following SARS-CoV-2 infection [56]. Another highlight of our data is the finding of a significant reduction in the testicular TGF-β1 levels. While increased TGF-β1 levels in the serum are in agreement with human clinical data and proposed to promote fibrosis [57], reduced levels of testicular TGF-β1, which regulates spermatogenesis and immune homeostasis pathways in the testis [58], may also be one of the underlying mechanisms of compromised testicular function in recovering COVID-19 patients.

Consistent with the clinical data [6,17,18], we also observed a decrease in the sperm count in mice, but this decrease was short-term after recovery from acute disease compared to humans, where 10–15% of recovering COVID-19 patients report lower sperm counts for almost 5–6 months [19]. However, whether this decline impacts male fertility remains unclear. Our fertility data are clinically important and suggest that the decline in male fertility correlates with significantly low sperm count and that the sperm count decline is reversed during the second spermatogenesis cycle phase in the seminiferous tubules after infection (spermatogenesis cycle is ~9 days in mouse). Though the mouse spermatogenesis cycle cannot be directly compared to the human cycle (~72 days), we speculate that severe COVID-19 in humans may likely compromise male fertility only in a subset of males during the first 2–3 spermatogenesis cycle phase after infection (2–4 months).

Combating systemic inflammation is beneficial in managing severe disease as well as long COVID. Dexamethasone is one of the drugs used to clinically manage inflammation in many diseases including COVID-19 [59]. Our data showing a significant impact of dexamethasone in preventing testicular injury strongly suggest that timely suppression of inflammation may facilitate the resolution of reproductive health complications in recovering males. However, the effects of a longer treatment may be needed to understand the ideal time course of the drug to prevent long-term decline in male reproductive health. As shown in the hypothetical model in Fig 6J, we propose that SARS-CoV-2-associated systemic cytokine storm triggers local testicular inflammation, which induces gross morphological alterations, disruption of the BTB, and recruitment of peripheral myeloid cells. These events manifest in the dysregulation of several fertility markers, many of which persist during recovery. While suppression of inflammation by dexamethasone mitigates testicular injury, the key inflammatory mediators driving the pathophysiology and underlying molecular pathways remain to be determined. In summary, our study improves the current understanding of the long-term implications of COVID-19 in the testis and supports the use of K18-hACE2 mice to evaluate different therapeutic strategies to prevent decline in male reproductive health in long COVID patients. Our findings also suggest the need for close monitoring of recovering COVID-19 patients to timely rescue the pathophysiological effects on male reproductive health.

## Methods

### Ethics statement

All experiments were performed on eight- to twelve-week-old males in accordance with the national and institutional guidelines for the care and use of animals after the University of Hawaii Institutional Animal Care and Use Committee (IACUC) approval. All SARS-CoV-2 infection experiments were conducted in animal Biosafety Level 3 facilities following the University of Hawaii’s Environmental Health and Safety Office plans. All personnel wore personal protective equipment, and all assays have been designed to conform to the safety requirements recommended by Biosafety in Microbiological and Biomedical Laboratories (BMBL), the US Department of Health and Human Services, the Public Health Service, the Centers for Disease Control and Prevention (CDC), and the National Institutes of Health (NIH).

### Infection of K18-hACE2 mice and virus quantitation

K18-hACE2 male mice (Jackson Laboratory Strain #034860**)** were infected intranasally with 10^4^ PFU of SARS-CoV-2 USA-HI-B.1.429, similar to the Delta strain of SARS-CoV-2 [60]. At least 5 independent sets of experiments were conducted to obtain tissues from at least 4 survivor mice at all time points. Mice were perfused with PBS, and tissues were either flash-frozen or fixed in 4% PFA and/or Bouin for various assays. SARS-CoV-2 titers and intracellular viral genome copies in the normalized tissue were measured by plaque assay and qRT-PCR respectively, as described by us previously [22]. Dexamethasone (Millipore Sigma) was administered intraperitoneally at 10mg/kg per mouse/day for 3 days starting day 2 post-infection.

### Testis histology analysis

For histology, either full testes or halved testes fixed in Bouin solution and embedded in paraffin were cut at 5 μm thickness and stained with Periodic-acid Schiff and hematoxylin (PAS-H). The stages of seminiferous tubules were identified based on the composition of cells near the basal membrane according to the method described by Ahmed and de Rooij as described [29,61]. For the analysis of seminiferous epithelium abnormalities, 50 tubules from each male were analyzed in a blinded manner and classified as normal or abnormal regarding their organizational and germ cell features. Individual histopathological alterations including cells in the lumen, lack of lumen, separation from the basal lamina, and specific germ cell defects were determined as described previously [29].

### RNA sequencing analysis and RT-PCR

RNA samples extracted from the whole testis from control and infected mice (n = 3/time point) were sent for sequencing using Illumina TruSeq stranded mRNA kit (sequencer: NovaSeq). Alignment was performed using STAR (v2.7.3a) aligner and analyzed as described previously [62]. Principal component analysis (PCA) was performed for normalized counts of all the protein-coding genes from all the samples. Differential expression analysis was performed using DESeq2. The number of significantly up or down differentially expressed protein-coding genes without fold change shrinkage in the treated group (n = 3/timepoint) vs. control group (n = 3) were used for the analyses (Significant differential expression cutoff: P value < 0.05 & log2 fold-change >|1|). The data quality check was performed using FastQC (v0.11.8). Gene Ontology Over Representation Analysis (ORA) was done using enrichGO method from clusterProfiler (OrgDb/ AnnotationDbi: org.Mm.eg.dbv3.7.0 for Mouse from Bioconductor). IPA was used to generate functional networks and predicted upstream transcriptional regulators. IPA cutoff criteria was set to log2 fold-change >|1| and p-value of <0.05 as described previously [62]. Polynomial regression (degree 2) was applied to model the gene expression trends from the existing time points (D3, D5, D8, D14, and D30) and predict gene expression levels at D40, D60, and D90. A cutoff of p<0.05 was applied and Log2 fold changes (log2fc) were calculated for the predicted time points relative to the control sample. CIBERSORTx, a machine learning tool, was used to analyze RNA-seq data to quantify the relative fractions of distinct cell type abundance and gene expression patterns as described by Wang et al [63]. Transcripts of select cell markers and cytokines were measured in the testis samples by qRT-PCR using specific primers, as shown in Table 1.

**Table 1:** List of qRT-PCR primers used in this study.

Gene	Gene ID	Forward Primer	Reverse Primer
SOX9	20682	5’- AGGAAGCTGGCAGACCAGTAC-3’	5’- TTGGAAGGCAGCGGTCGA-3’
GATA4	14463	5’- GGTTACCCAGGCACAGATGA-3’	5’- GCCACAGTGGTGGTGAAATC-3’
STAR	20844	5’- GAATCCTGGGCAGAGTGGAC-3’	5’- GGGAAGTGGCTTGTGAAGGT-3’
CYP11A1	13070	5’- ACTGCCTGCTTGTCAAGGTG-3’	5’- GTGTAGGCATGAGGGGTAGG-3’
ZBTB16	22637	5’- TGAAGCCGACACTGTGACAA-3’	5’- CCCTTCATTCGACAGTTCCT-3’
SOX2	20674	5’- CGCCGAGTGGAAACTTTTGTCC-3’	5’- GGAGTGGGAGGAAGAGGTAACC-3’
IL6	16193	5’- GTTCTCTGGGAAATCGTGGA-3’	5’- TGTACTCCAGGTAGCTATGG-3’
TNFA	21926	5’- CATCTTCTCAAAATTCGAGTGACAA-3’	5’- TGGGAGTAGACAAGGTACAACCC-3’

### Immunofluorescence and TUNEL assays

PFA-fixed tissues from mock and infected K18-hACE2 mice were processed in sucrose and embedded in OCT. Sections (10μm thickness) were permeabilized with 0.1% Triton X-100, blocked with 5% bovine serum albumin and stained with primary antibodies against anti-CD68 (Invitrogen, 1:250) and anti-CD11b (Invitrogen, 1:250), followed by fluorophore-conjugated secondary antibody (Invitrogen Alexa Fluor 594-conjugated, at 1:1000). TUNEL assay was performed using the Invitrogen Click-iT Plus TUNEL Assay Kit (Thermo Fisher Sci). ImageJ/Fiji software was used for densitometry of all the representative images using the average of 3 different fields from 3 different mice per group.

### Multi-plex and single-plex Enzyme-Linked immunosorbent assays

Lung, heart, and testis tissue lysates prepared in tissue extraction reagent with 1X protease and phosphatase inhibitors were used for ELISA of TGF-β1 Mouse ELISA Kit (Thermo Fisher Sci), Mouse Testosterone ELISA Kit (Crystal Chem), Mouse FSH ELISA Kit (Abclonal), Mouse INHB ELISA kit (Abclonal), Mouse LH ELISA Kit (Abclonal). MILLIPLEX Mouse Cytokine/Chemokine Magnetic Bead Panel (Millipore Sigma MCYTOMAG-70K-PMX) was used to measure 27 cytokines and chemokines as described previously [64].

### Epididymal sperm count and fertility assays

Cauda epidydimal sperm from each mouse were released into 1 mL of PBS and incubated for 30 min at 37°C. Spermatozoa were extracted by filtering through 70 μm filters, and the total sperm numbers were counted. Fertility was tested for 5 recovered males who were proven fertile before infection. At D9 post-infection, each male was placed in a new cage with two females for 6 days, and females were observed daily for vaginal plugs to confirm mating. At D15, males were sacrificed to measure sperm count, and the females were sacrificed 10 days after separating from males to assess implantation sites reflective of embryo count. The male was considered fertile if the pregnancy was established in at least one of the 2 females.

### Statistical analysis

SARS-CoV-2 titers are reported as means +/- standard error of the mean (SEM) of data from ≥3 independent experiments. The statistically significant differences between data from different groups were determined by unpaired Student’s t-test or Mann-Whitney test using GraphPad Prism software (version 10.0.2). Pearson’s correlations, and matrices were performed using GraphPad Prism software. Pearson correlation coefficient >|0.6| was considered statistically significant.

## Supporting information

S1 Fig SARS-CoV-2 infection of K18-hACE2 mice. Mice were infected with 10^4^ log PFU of the virus intranasally and **(A)** Survival curve and **(B)** Percent weight change compared to uninfected controls were evaluated. **(C)** SARS-CoV-2 genome copies in the lung, heart, and testis at indicated time points measured using qRT-PCR and expressed as copies/mg RNA. **(D)** Plaque assay of progeny SARS-CoV-2 titers in the tissue homogenates of lung, heart, and testis measured at indicated time points. Significance *p<0.05, **p<0.01, ***p<0.001, ****p<0.0001 was determined using student’s t-test.
(TIFF)

S2 Fig Germ cell defect analysis. Testes from male K18-hACE mice infected with SARS-CoV-2 were evaluated at pre-symptomatic (D3), symptomatic (D5), short-term recovery (D8-14) and long-term recovery (D30) stages, with testes from uninfected mice served as controls (Ctrl). Nuclear morphology, nucleosomal patterning, cell size, cell location, and cytoplasmic appearance were used to also identify germ cell abnormalities. Five different germ cell abnormality types were differentiated. **(A)** Representative images of **AC**, apoptotic cell; **AM**, apoptotic cell at meiotic metaphase, **DN**, cell with degenerating nucleus; **ARB**, abnormal residual body; and **O**, all other abnormalities. **AC** usually has a larger size than expected for the given cell. The cytoplasm appears pink to fuchsia in color and the nucleus can have a glossy or pale appearance of uniform ungranulated consistency, or a ground glass appearance due to mottled dark splotches. **AM** exhibits features of an apoptotic cell, but the nucleus has a clear metaphase plate. Metaphase plate orientation may vary, which may result in appearance variability. **DN** has a turbid to opaque appearance, or there may also be areas that are wispy. The nucleus size is frequently larger than usual for the cell type in which it is found, and the surrounding cytoplasm looks like a white halo giving an overall expression of the cell “ballooning” around the nucleus. **ARB** represent a deviation of naturally occurring residual bodies (RB), which are byproducts of spermiogenesis made of cytoplasm shed as spermatids elongate. RB appear purple when stained with PAS-H and lack nuclear content which distinguishes them from apoptotic cells. Their presence is stage-specific and restricted to stage VII-VIII and IX-X. RB are expected to be phagocytized by the Sertoli cells following stage VII-VIII. When present in later stages, they are considered ABR. **O** include vacuoles (characterized as areas of the seminiferous epithelium lacking germ cells causing an open space to form within the perimeter of the Sertoli cells) and cell remnants (small, fuchsia-colored bodies lacking nucleus that are not stage dependent and are much smaller than residual bodies). Because vacuoles and cell remnants appeared infrequently, they were grouped together as one category. **(B)** Quantification of seminiferous tubule defects. For each male, 50 tubules were examined. Scale, 100 μm. Each data point corresponds to a different mouse.
(TIFF)

S3 Fig Gene ontology (GO) overrepresentation analysis (ORA) of testis from SARS-CoV-2-infected mice. **(A)** Top downregulated gene pathways were identified at D3, -5, -8, -14, and 30 compared to control testis (left column). **(B)** Top upregulated gene pathways were identified at D3, -5, -8, -14, and 30 compared to control testis (right column). Cutoffs for all analyses were p<0.05 and log2fc ≥∣1∣
(TIFF)

S4 Fig Ingenuity pathway analysis of upstream regulators reveals significant alterations in fertility and cell death markers following SARS-CoV-2 infection of K18hACE2 mice. **(A)** z-score of individual select upstream regulators associated with fertility at indicated time points. The mRNA fold change of genes targeted by **(B)** GATA6 and **(C)** AR at indicated time points in the testis from infected mice and expressed as log2fc.
(TIFF)

S5 Fig (A) Cell type proportions in mouse testis at different time points post SARS-CoV-2 infection compared to uninfected controls (Ctrl). Cell type proportions in the RNA seq data were estimated using CIBERSORTx deconvolution analysis. The proportion of each cell type is color-coded as follows: Spermatogonia cells (pink), Sertoli cells (orange), Leydig cells (red), Peritubular myoid cells (brown), Macrophages (blue), Neutrophils (green), Dendritic cells (grey), and T cells (maroon). Validation of CIBERSORTx results in testis RNA samples using qRT-PCR analysis of cell type-specific marker genes (B) Sertoli cells (GATA4, SOX9), (C) Leydig cells (STAR, CYP11A1) and (D) Spermatogonia cells (ZBTB16, SOX2). Gene expression is shown as mRNA fold change relative to uninfected controls. (E) log2fc of genes associated with gap junction, tight junction, and filament molecules at D5 (left) and D8 (right) in the testis. The dotted line represents cutoff (log2fc>∣1∣, p <0.05).
(TIFF)

S6 Fig **(A)** Heatmap displaying z-scores of top dysregulated pathways and select diseases and functions in the existing (D3, D5, D8, D14, and D30) and extrapolated (D40, D60, D90) timepoints. Gene expression for predicted time points was estimated using polynomial regression (degree 2) based on the expression data from Days 3, 5, 8, 14, and 30. (upregulated, orange and downregulated, purple). **(B-C)** Heatmaps showing extrapolated changes in the expression of individual genes involved in **(B)** integrin-linked kinase (ILK) signaling and **(C)** focal adhesion kinase (FAK) signaling pathways at each time point. **(D)** The heatmap of differential upstream regulator expression across indicated time points (log2fc>∣1∣, p <0.05).
(TIFF)

S7 Fig Age-matched male survivors were mated with females (2 females per male) starting at D14 and then separated after 6 days. The pregnancy was evaluated by counting the embryos after 10 days. Relationship between the percent change in the sperm count/epididymis (left y-axis, circle, blue) in each male after 6 days of mating and the number of fetuses (right y-axis, triangle, red) in each female (n, two females for each male).
(TIFF)

S8 Fig Quantification of seminiferous tubule organizational and germ cell defects in dexamethasone-treated mice following SARS-CoV-2 infection. For each male, 50 tubules were examined and classified as normal or abnormal with regard to their organizational and germ cell features as explained in S2 Fig. Each data point corresponds to a different mouse. D5 data are the same as shown in Figs 1 and S2 and are shown here for visual comparison with Dex-D5. Statistical significance (t-test, Ctrl vs. Dex-D5). *p<0.05. The differences approaching significance (p = 0.05–0.1) are shown directly in the graph.
(TIFF)
