# Supplementary figures and images for: SARS-CoV-2-induced cytokine storm drives prolonged testicular injury and functional impairment in mice that are mitigated by dexamethasone
Source: PLoS Pathog. 2025 Jan 7;21(1):e1012804. doi: 10.1371/journal.ppat.1012804 (PMC11706467; doi:10.1371/journal.ppat.1012804)

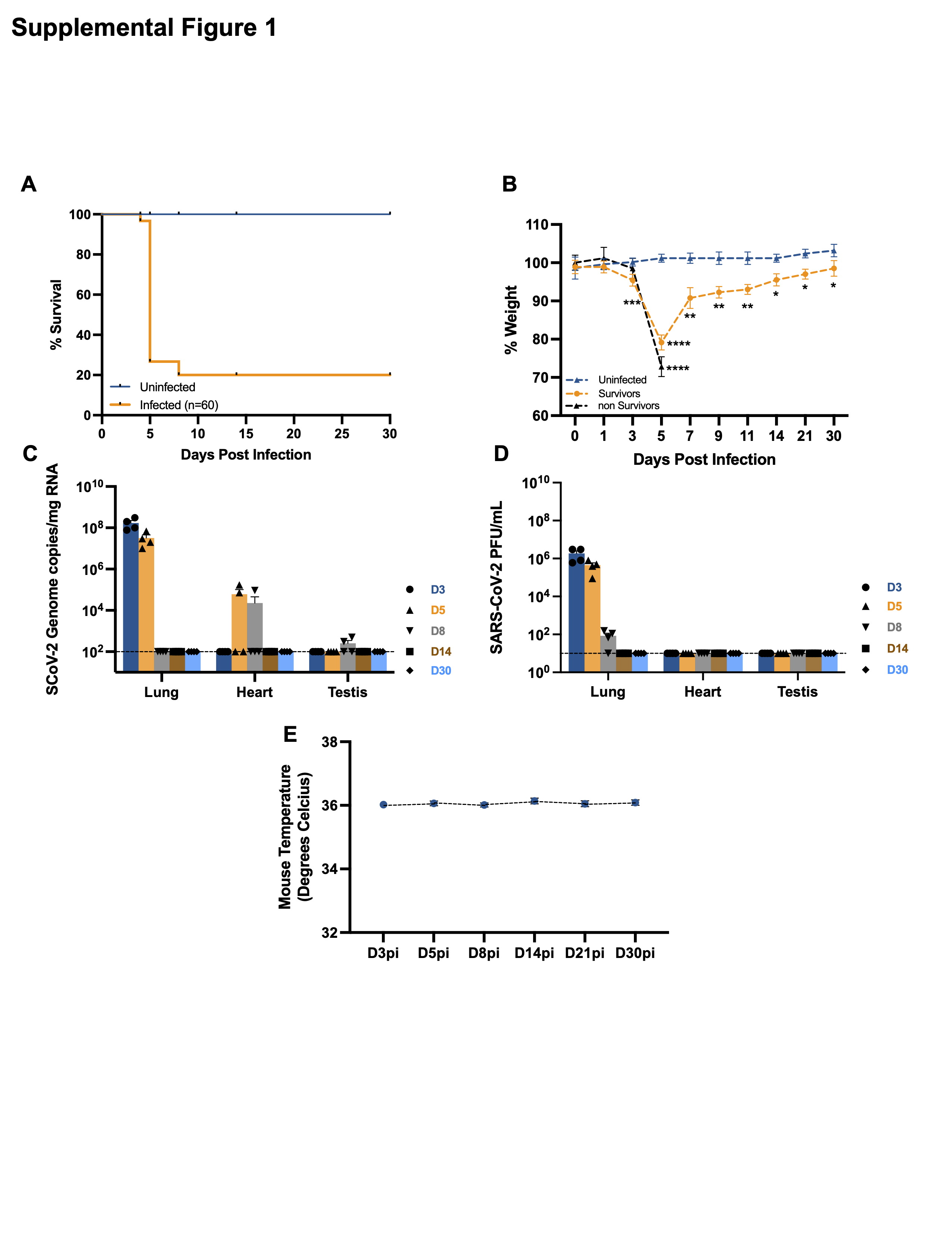

Supplement: S1 Fig — Mice were infected with 104 log PFU of the virus intranasally and (A) Survival curve and (B) Percent weight change compared to uninfected controls were evaluated. (C) SARS-CoV-2 genome copies in the lung, heart, and testis at indicated time points measured using qRT-PCR and expressed as copies/mg RNA. (D) Plaque assay of progeny SARS-CoV-2 titers in the tissue homogenates of lung, heart, and testis measured at indicated time points. Significance *p<0.05, **p<0.01, ***p<0.001, ****p<0.0001 was determined using student’s t-test. (TIFF) [file ppat.1012804.s001.tiff]

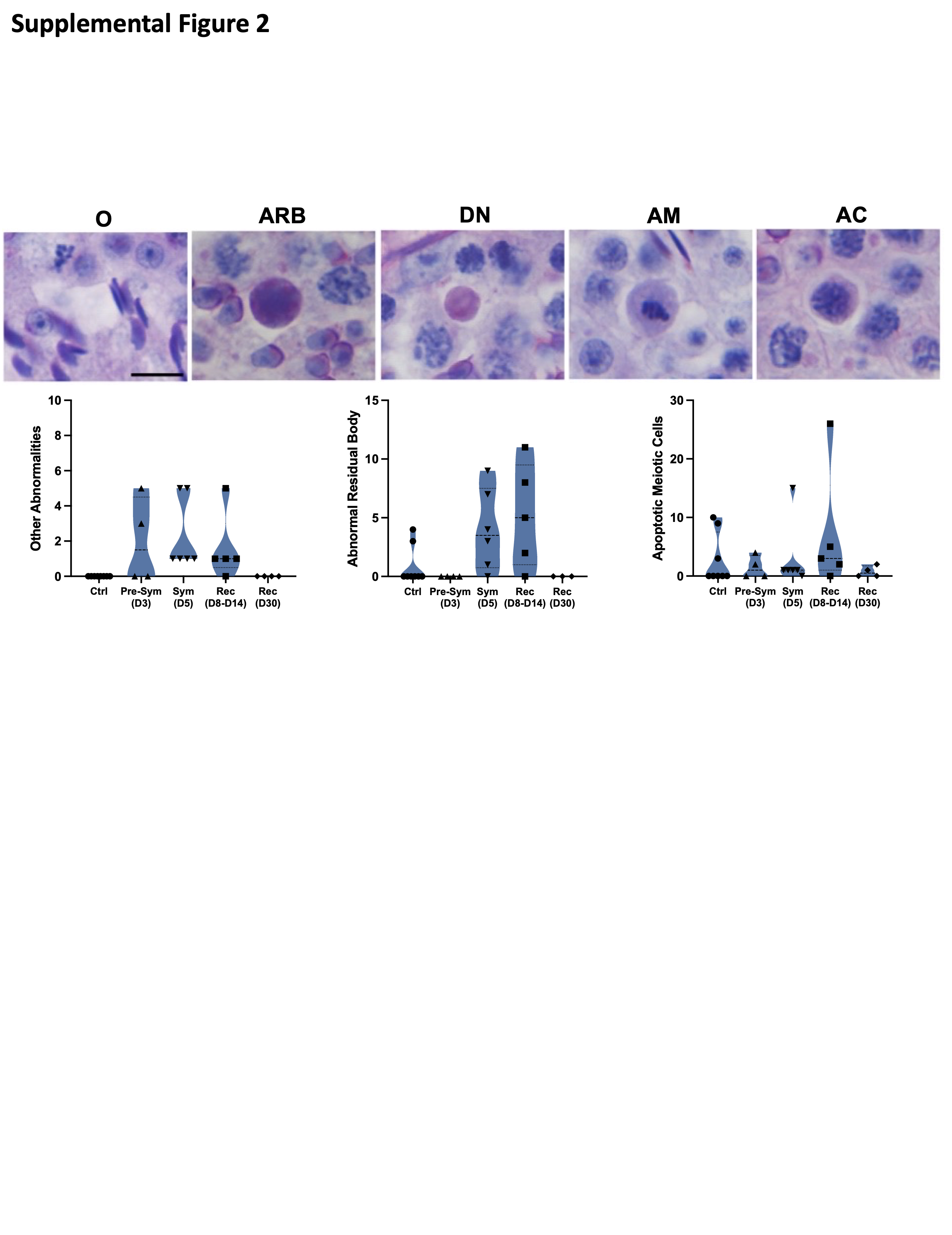

Supplement: S2 Fig — Testes from male K18-hACE mice infected with SARS-CoV-2 were evaluated at pre-symptomatic (D3), symptomatic (D5), short-term recovery (D8-14) and long-term recovery (D30) stages, with testes from uninfected mice served as controls (Ctrl). Nuclear morphology, nucleosomal patterning, cell size, cell location, and cytoplasmic appearance were used to also identify germ cell abnormalities. Five different germ cell abnormality types were differentiated. (A) Representative images of AC, apoptotic cell; AM, apoptotic cell at meiotic metaphase, DN, cell with degenerating nucleus; ARB, abnormal residual body; and O, all other abnormalities. AC usually has a larger size than expected for the given cell. The cytoplasm appears pink to fuchsia in color and the nucleus can have a glossy or pale appearance of uniform ungranulated consistency, or a ground glass appearance due to mottled dark splotches. AM exhibits features of an apoptotic cell, but the nucleus has a clear metaphase plate. Metaphase plate orientation may vary, which may result in appearance variability. DN has a turbid to opaque appearance, or there may also be areas that are wispy. The nucleus size is frequently larger than usual for the cell type in which it is found, and the surrounding cytoplasm looks like a white halo giving an overall expression of the cell “ballooning” around the nucleus. ARB represent a deviation of naturally occurring residual bodies (RB), which are byproducts of spermiogenesis made of cytoplasm shed as spermatids elongate. RB appear purple when stained with PAS-H and lack nuclear content which distinguishes them from apoptotic cells. Their presence is stage-specific and restricted to stage VII-VIII and IX-X. RB are expected to be phagocytized by the Sertoli cells following stage VII-VIII. When present in later stages, they are considered ABR. O include vacuoles (characterized as areas of the seminiferous epithelium lacking germ cells causing an open space to form within the perimeter of the [file ppat.1012804.s002.tiff]

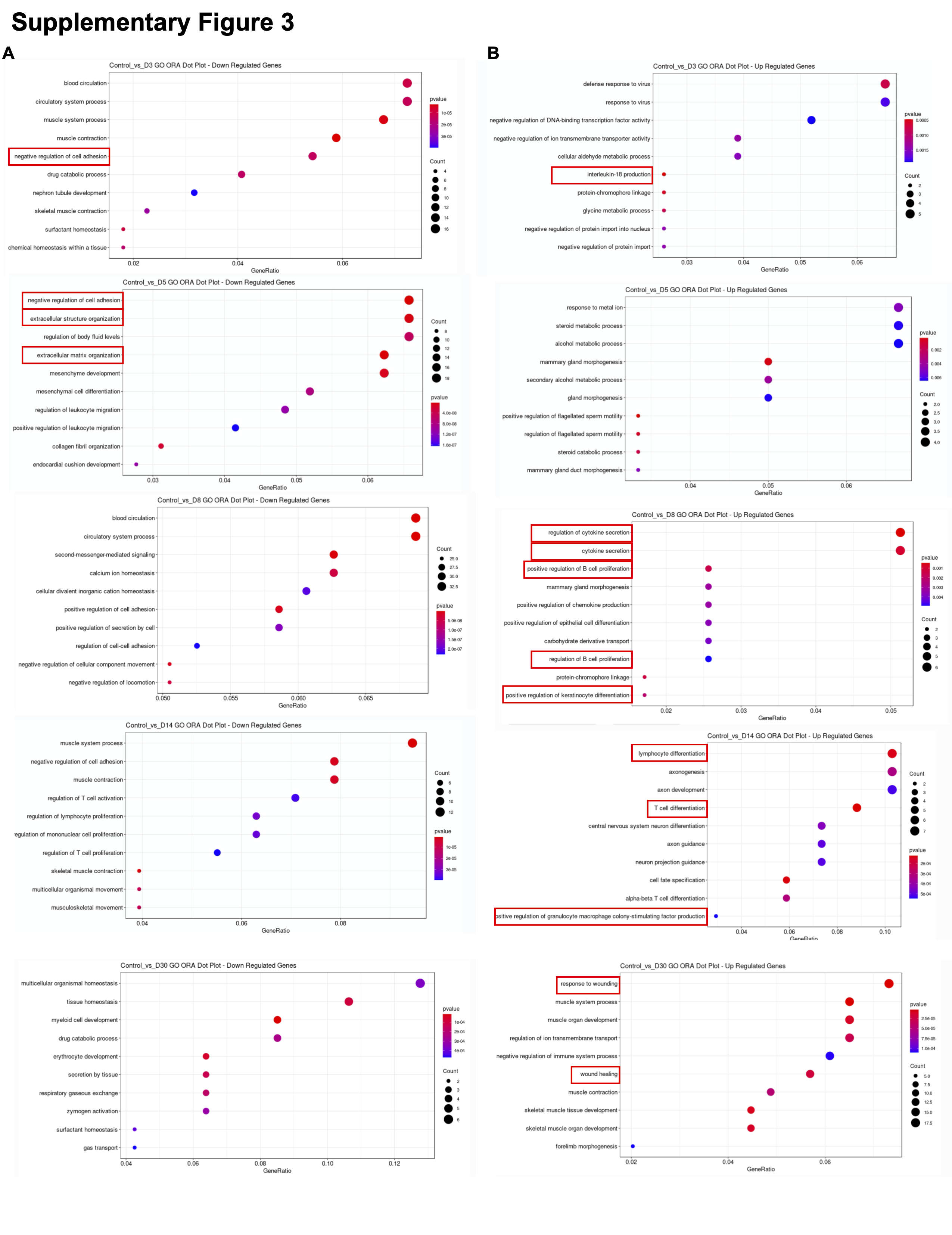

Supplement: S3 Fig — (A) Top downregulated gene pathways were identified at D3, -5, -8, -14, and 30 compared to control testis (left column). (B) Top upregulated gene pathways were identified at D3, -5, -8, -14, and 30 compared to control testis (right column). Cutoffs for all analyses were p<0.05 and log2fc ≥∣1∣ (TIFF) [file ppat.1012804.s003.tiff]

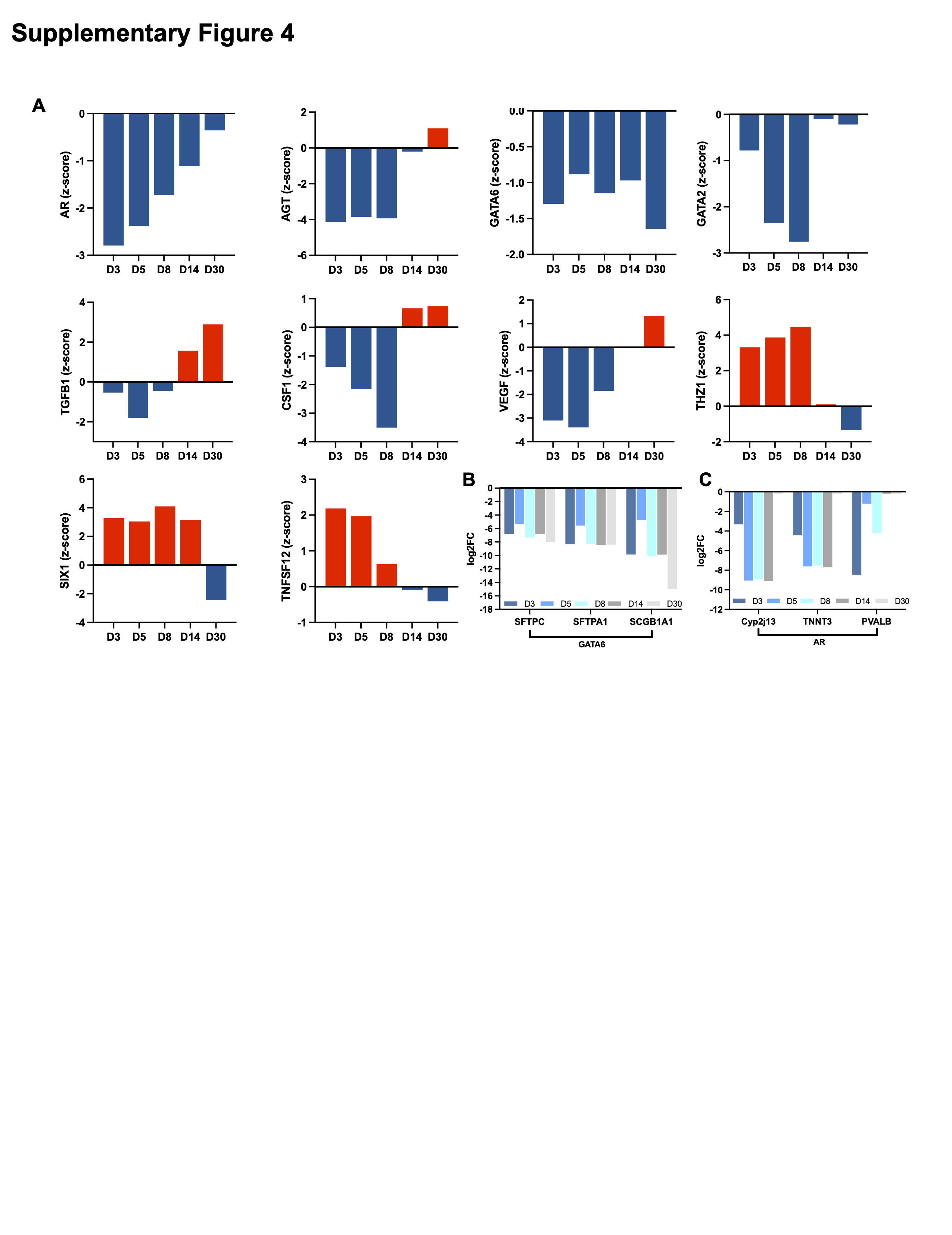

Supplement: S4 Fig — (A) z-score of individual select upstream regulators associated with fertility at indicated time points. The mRNA fold change of genes targeted by (B) GATA6 and (C) AR at indicated time points in the testis from infected mice and expressed as log2fc. (TIFF) [file ppat.1012804.s004.tiff]

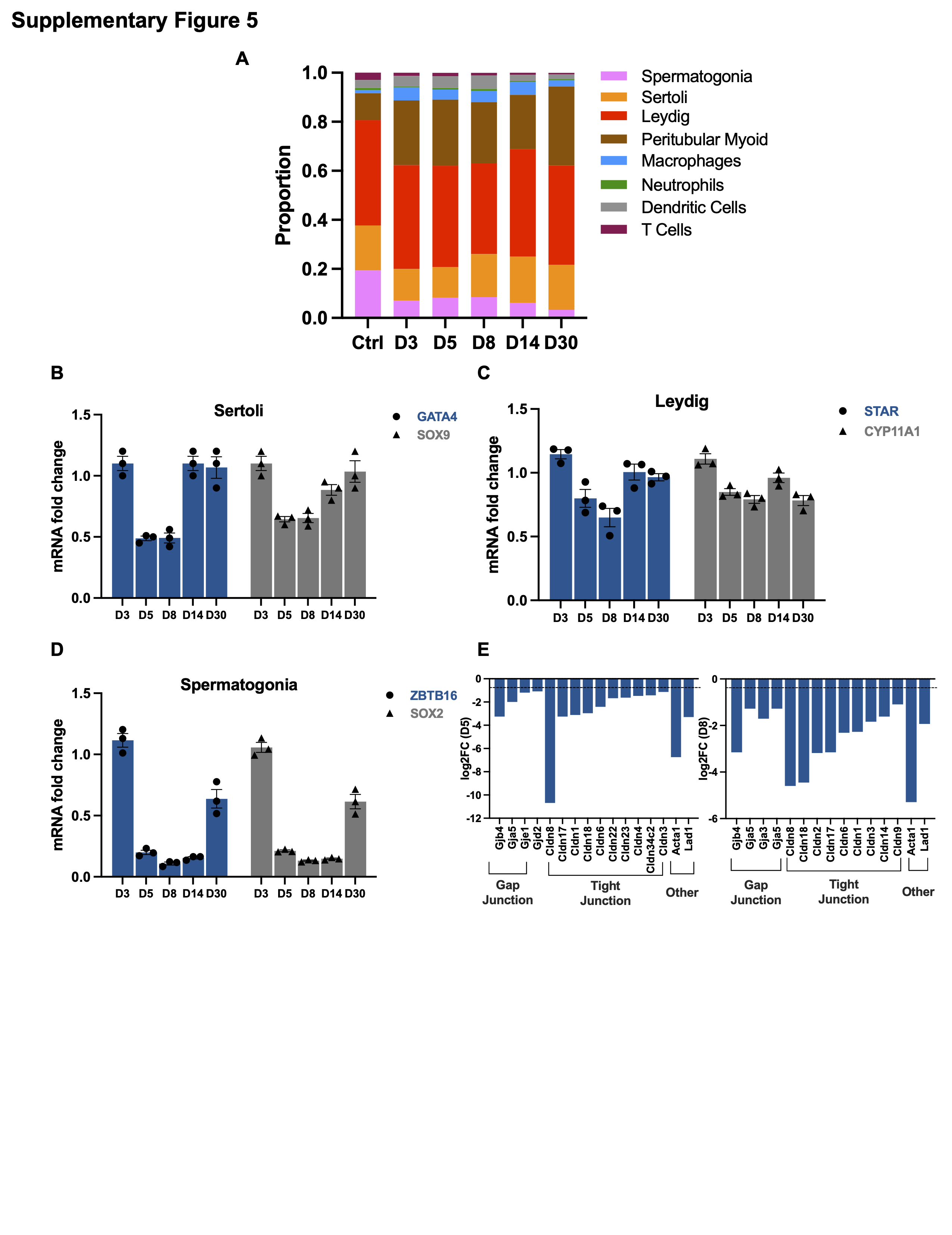

Supplement: S5 Fig — (A) Cell type proportions in mouse testis at different time points post SARS-CoV-2 infection compared to uninfected controls (Ctrl). Cell type proportions in the RNA seq data were estimated using CIBERSORTx deconvolution analysis. The proportion of each cell type is color-coded as follows: Spermatogonia cells (pink), Sertoli cells (orange), Leydig cells (red), Peritubular myoid cells (brown), Macrophages (blue), Neutrophils (green), Dendritic cells (grey), and T cells (maroon). Validation of CIBERSORTx results in testis RNA samples using qRT-PCR analysis of cell type-specific marker genes (B) Sertoli cells (GATA4, SOX9), (C) Leydig cells (STAR, CYP11A1) and (D) Spermatogonia cells (ZBTB16, SOX2). Gene expression is shown as mRNA fold change relative to uninfected controls. (E) log2fc of genes associated with gap junction, tight junction, and filament molecules at D5 (left) and D8 (right) in the testis. The dotted line represents cutoff (log2fc>∣1∣, p <0.05). (TIFF) [file ppat.1012804.s005.tiff]

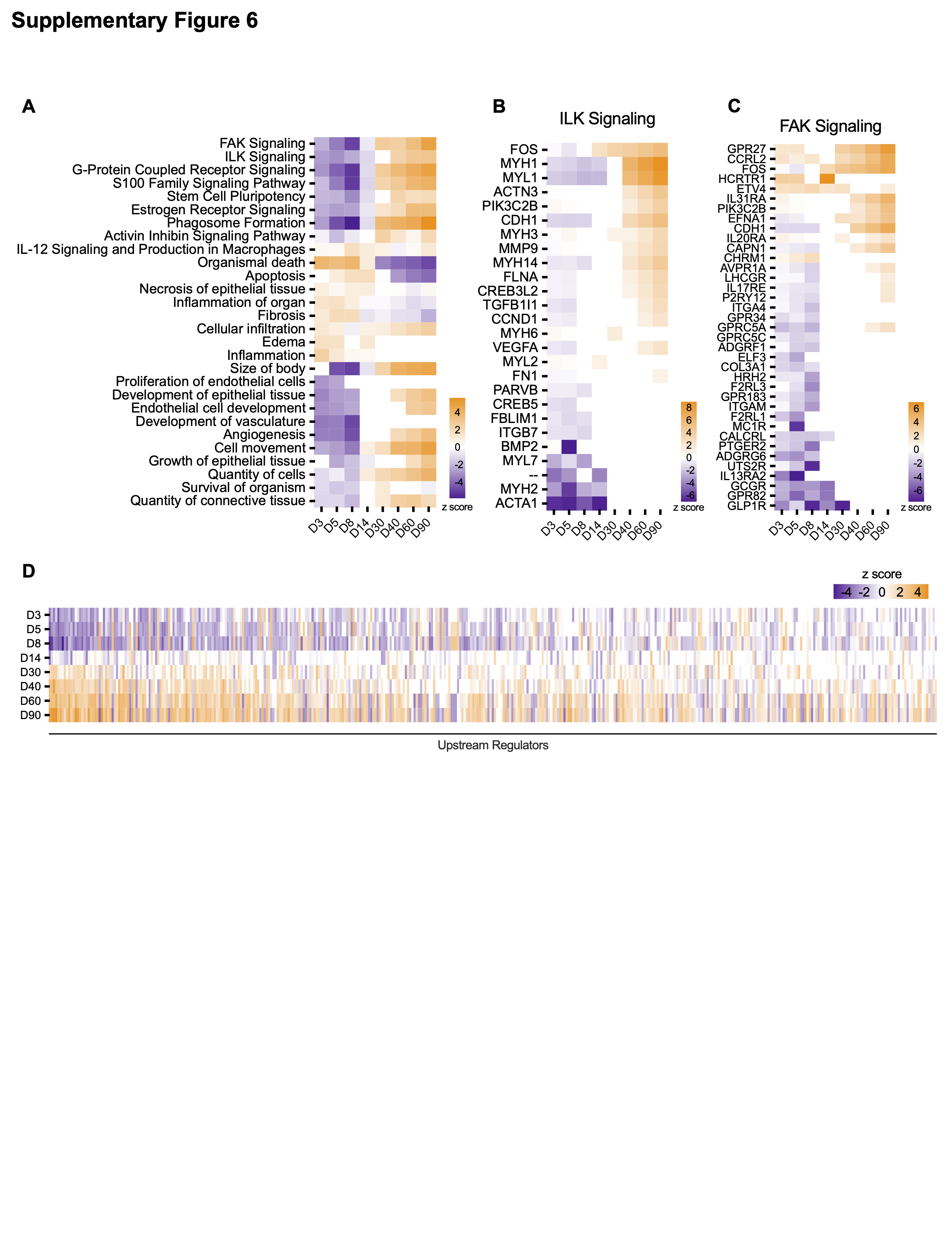

Supplement: S6 Fig — (A) Heatmap displaying z-scores of top dysregulated pathways and select diseases and functions in the existing (D3, D5, D8, D14, and D30) and extrapolated (D40, D60, D90) timepoints. Gene expression for predicted time points was estimated using polynomial regression (degree 2) based on the expression data from Days 3, 5, 8, 14, and 30. (upregulated, orange and downregulated, purple). (B-C) Heatmaps showing extrapolated changes in the expression of individual genes involved in (B) integrin-linked kinase (ILK) signaling and (C) focal adhesion kinase (FAK) signaling pathways at each time point. (D) The heatmap of differential upstream regulator expression across indicated time points (log2fc>∣1∣, p <0.05). (TIFF) [file ppat.1012804.s006.tiff]

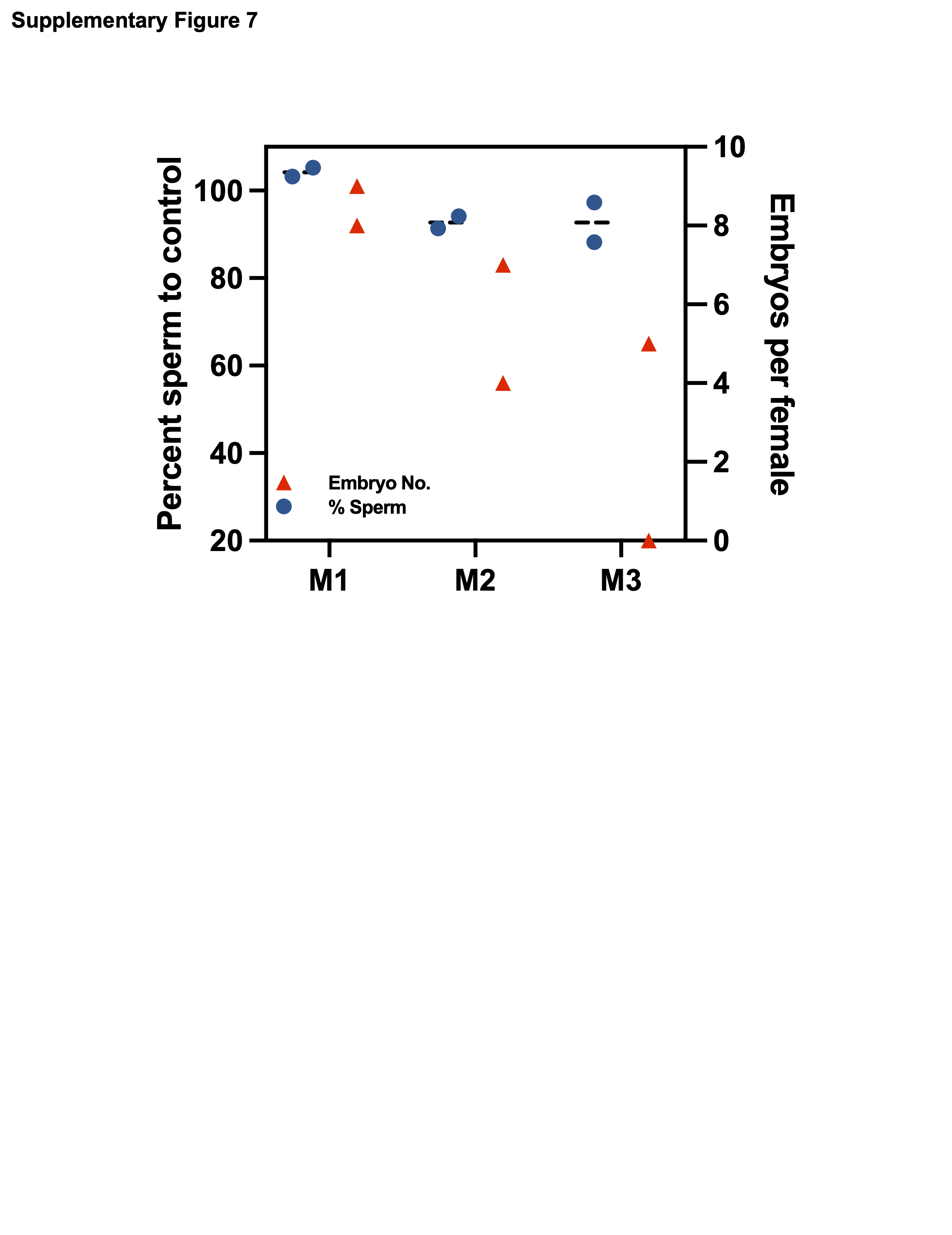

Supplement: S7 Fig — The pregnancy was evaluated by counting the embryos after 10 days. Relationship between the percent change in the sperm count/epididymis (left y-axis, circle, blue) in each male after 6 days of mating and the number of fetuses (right y-axis, triangle, red) in each female (n, two females for each male). (TIFF) [file ppat.1012804.s007.tiff]

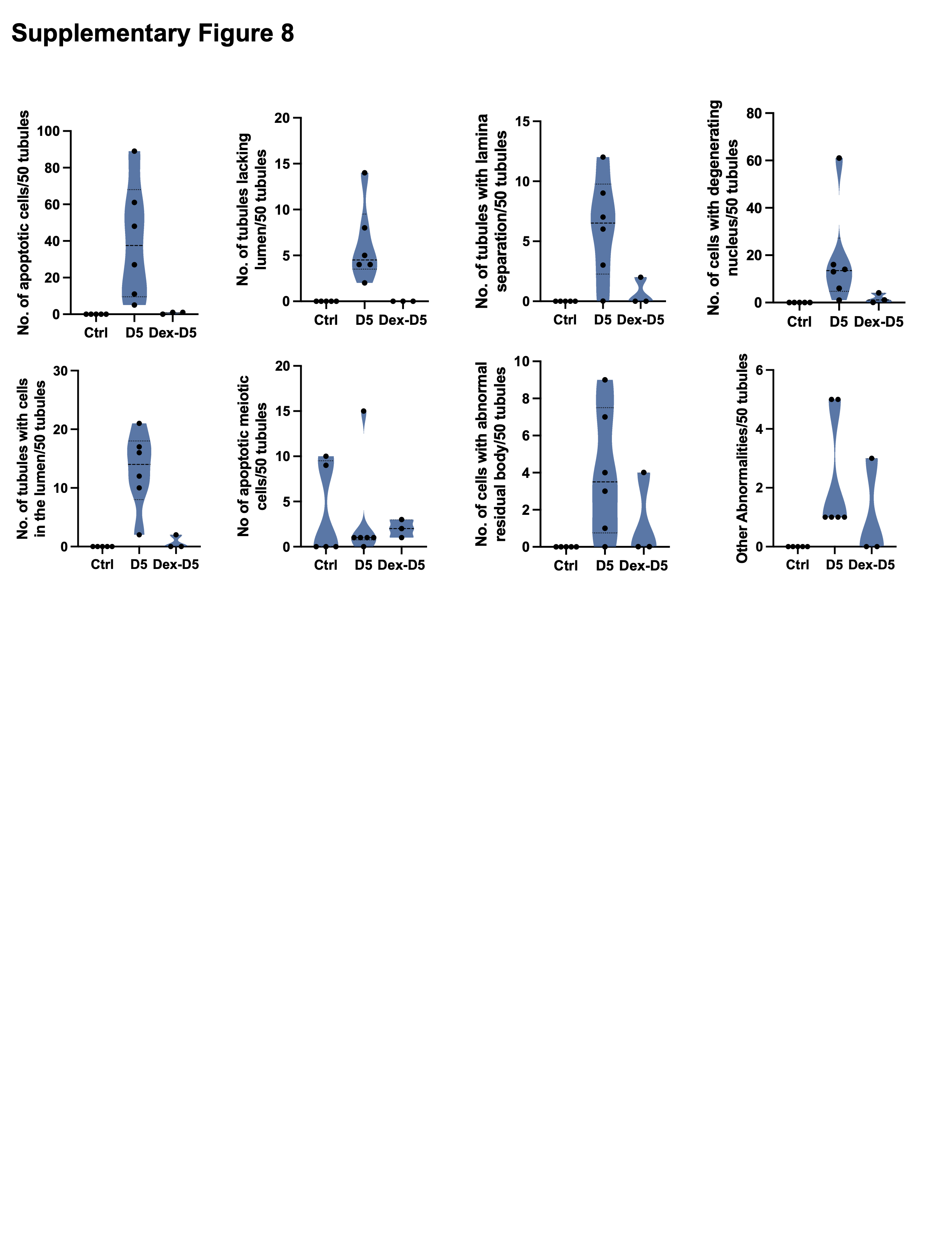

Supplement: S8 Fig — For each male, 50 tubules were examined and classified as normal or abnormal with regard to their organizational and germ cell features as explained in S2 Fig. Each data point corresponds to a different mouse. D5 data are the same as shown in Figs 1 and S2 and are shown here for visual comparison with Dex-D5. Statistical significance (t-test, Ctrl vs. Dex-D5). *p<0.05. The differences approaching significance (p = 0.05–0.1) are shown directly in the graph. (TIFF) [file ppat.1012804.s008.tiff]
